# Mechanisms of palmitic acid-conjugated antisense oligonucleotide distribution in mice

**DOI:** 10.1093/nar/gkaa164

**Published:** 2020-03-17

**Authors:** Alfred E Chappell, Hans J Gaus, Andres Berdeja, Ruchi Gupta, Minji Jo, Thazha P Prakash, Michael Oestergaard, Eric E Swayze, Punit P Seth

**Affiliations:** Ionis Pharmaceuticals, Inc. 2855 Gazelle Court, Carlsbad, CA 92010, USA

## Abstract

Conjugation of antisense oligonucleotide (ASO) with a variety of distinct lipophilic moieties like fatty acids and cholesterol increases ASO accumulation and activity in multiple tissues. While lipid conjugation increases tissue exposure in mice and reduces excretion of ASO in urine, histological review of skeletal and cardiac muscle indicates that the increased tissue accumulation of lipid conjugated ASO is isolated to the interstitium. Administration of palmitic acid-conjugated ASO (Palm-ASO) in mice results in a rapid and substantial accumulation in the interstitium of muscle tissue followed by relatively rapid clearance and only slight increases in intracellular accumulation in myocytes. We propose a model whereby increased affinity for lipid particles, albumin, and other plasma proteins by lipid-conjugation facilitates ASO transport across endothelial barriers into tissue interstitium. However, this increased affinity for lipid particles and plasma proteins also facilitates the transport of ASO from the interstitium to the lymph and back into circulation. The cumulative effect is only a slight (∼2-fold) increase in tissue accumulation and similar increase in ASO activity. To support this proposal, we demonstrate that the activity of lipid conjugated ASO was reduced in two mouse models with defects in endothelial transport of macromolecules: caveolin-1 knockout (Cav1^−/−^) and FcRn knockout (FcRn^−/−^).

## INTRODUCTION

Antisense oligonucleotides are effective therapeutic agents for treating a variety of diseases with several approved for use in the clinic and many more in various stages of clinical development (1). Systemically administered ASO are distributed primarily to liver, kidney and spleen through kinetics that are largely driven by the phosphorothioate backbone that provides resistance to nuclease metabolism and enhances association with plasma and cell-surface proteins (2,3). While systemic administration of ASO can achieve robust mRNA knockdown in additional tissues like skeletal muscle, cardiac muscle, lung, and various tumors, relatively high doses are required (4,5). Thus, it will be therapeutically valuable to improve activity in these tissues to better address clinical needs.

Strategies to improve ASO activity include targeted delivery of ASO to specific tissues or cell types through direct conjugation with small molecules, carbohydrates, peptides, proteins, or antibody ligands that target cell-surface acceptor proteins in tissues of interest (6). Perhaps the most successful example of targeted ASO delivery is the 10–60-fold enhanced activity observed in hepatocytes with ASO conjugated with a triantennary *N*-acetyl galactosamine (GalNAc) through ASGR-mediated internalization (7). Another promising delivery ligand is Glucagon-like peptide 1 (GLP-1) which improved ASO activity in pancreatic B-cells by >50-fold over unconjugated ASO (8).

We and others demonstrated that conjugating hydrophobic moieties like fatty acids, cholesterol, and tocopherol can improve ASO activity in skeletal and cardiac muscle of rodents and non-human primates (9–12). We also showed that conjugating these hydrophobic moieties enhances association with plasma proteins such as albumin as well as lipoproteins such as LDL and HDL. Albumin is the most abundant plasma protein and performs several important physiological functions. About 60% of albumin is extravascular and it is estimated that albumin makes approximately 28 trips through the lymphatic system and returning to circulation in its lifetime (∼19 days *t*_1/2_) in humans (13). Albumin is transported across the continuous capillary endothelium by caveolin-1 (Cav1) mediated transcytosis (14,15). In addition, albumin is rescued from lysosomal degradation after internalization into cells by FcRn mediated exocytosis which binds albumin more tightly at the acidic pH in endosomes as compared to neutral pH environment of extracellular fluids (16). However, it was not clear why enhancing association with plasma proteins could enhance ASO activity in muscle tissues. To this end, we interrogated a hypothesis that conjugating ASO with palmitic acid (Palm-ASO) increases ASO activity in skeletal and cardiac muscle by facilitating ASO delivery across the continuous capillary endothelium in these tissues. As part of this effort, we examined the kinetics of distribution and activity of Palm-ASO in Cav1^−/−^, FcRn^−/−^ and Alb^−/−^ mice. We found that while the Palm-ASO showed reduced activity in Cav1^−/−^ and FcRn^−/−^ mice, it surprisingly showed increased activity in Alb^−/−^ mice suggesting that enhanced association with plasma proteins can facilitate distribution but impede cellular entry into parenchymal cells. Our results provide insights into how ASO traverse tissue barriers and suggest that complementing lipid-conjugation with cell-targeting ligands may provide even greater enhancements in ASO potency in tissues such as cardiac and skeletal muscle

## MATERIALS AND METHODS

### Antisense oligonucleotides

Antisense oligonucleotides targeting MALAT1 RNA with and without palmitic acid conjugation were used for the studies described in this report. The nucleotide sequence and chemistry were the same for both fully phosphorothioate oligonucleotides: GCATTCTAATAGCAGC, with three (*S*)-constrained ethyl (cEt) BNA nucleotides at each terminus (underlined) and ten DNA nucleotides in the middle (17). Palmitic acid was conjugated at the 5′-terminus of the ASO with a phosphodiester linkage (10). In some experiments, an unconjugated negative-control ASO (Control ASO) without any perfect-matched RNA target was used: CGCCGATAAGGTACAC. All ASO were synthesized using a procedure described previously (10).

### Care, treatment, and sample collection of mice

All animal experiments involving mice were conducted at Ionis Pharmaceuticals, Inc. in accordance with the American Association for the Accreditation of Laboratory Animal Care (AAALAC) guidelines and under protocols approved by the Animal Welfare Committee (Cold Spring Harbor Laboratory's Institutional Animal Care and Use Committee guidelines). Test articles (ASO or Palm-ASO) were administered by subcutaneous injection at the nape. At tissue collection times or study termination, mice were anesthetized using isoflurane, blood was collected by cardiac puncture exsanguination with K2-EDTA (Becton Dickinson Franklin Lakes, NJ, USA) and mice were euthanized by cervical dislocation. Plasma was separated by centrifugation at 10,000 rcf for 4 min at 4°C. Tissues were collected at necropsy, weighed, flash frozen on liquid nitrogen and stored at −60°C, or immediately homogenized in guanidine isothiocyanate with 2% β-mercaptoethanol.

**Figure 1.**
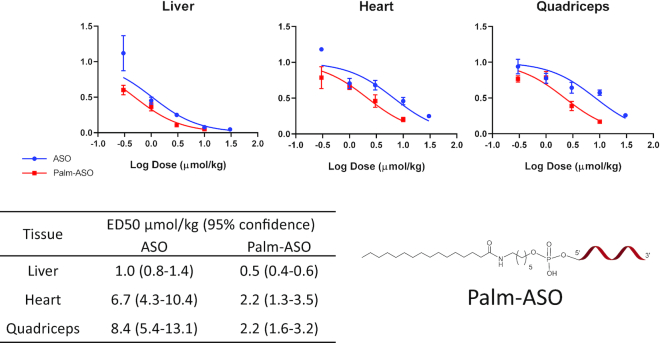
Conjugation of ASO with palmitic acid potentiates target mRNA knockdown in several tissues. A 3–10–3 PS cEt ASO (GCATTCTAATAGCAGC) targeted to MALAT1 or the same ASO conjugated at the 5′ terminus with palmitic acid was administered subcutaneously to male C57Bl/6 mice at concentrations ranging from 0.3 to 30 μmol/kg. Mice were terminated 72 h after administration and MALAT1 mRNA was extracted from liver, heart and quadricep tissues and quantified by qPCR. The ED50 is lowered 3–4-fold in heart and quadriceps.

In the first experiment comparing activities of ASO and Palm-ASO (Figure 1), male C57Bl/6J (BL6) mice (*n* = 4/test article/dose) were subcutaneously administered 0.3, 1, 3, 10 or 30 μmol/kg of ASO or Palm-ASO; study was terminated 72 hours after treatment; liver, heart and quadriceps were collected; and MALAT1 RNA expression normalized to cyclophilin in tissues was quantified using qRT-PCR.

**Figure 2.**
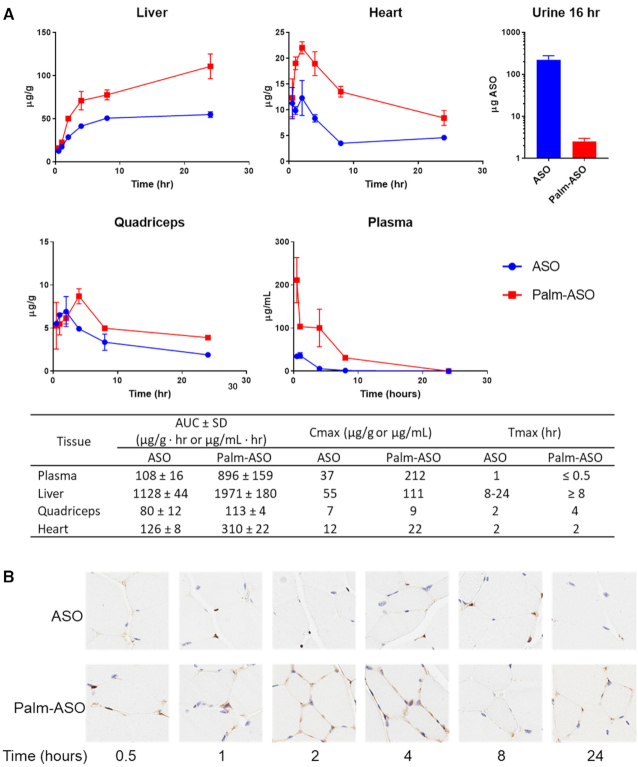
Conjugation of ASO with palmitate increases plasma and tissue AUC. Palm-ASO or ASO was administered subcutaneously to BL/6 mice at 7.5 μmol/kg and tissues collected from 0.5 to 24 h. (**A**) Palm-conjugated ASO has slower clearance from plasma, less excretion in urine, and greater accumulation in many tissues compared with unconjugated ASO. (**B**) Sections of quadricep muscle were stained with eosin (blue) and immunostained with rabbit anti-PS-ASO serum (brown). From 0.5 to 4 h, ASO staining appears darker in tissues from animals dosed with Palm-ASO compared with ASO and is concentrated along intercellular boundaries, suggesting ASO primarily occupies interstitial space or plasma membrane. At 8–24 h, punctate foci within the cells suggests internalization of the ASO.

In comparing early distribution of ASO and Palm-ASO (Figure 2), male C57Bl/6J mice (*n* = 4/test article/collection time), were administered 7.5 μmol/kg ASO or Palm-ASO subcutaneously and plasma (cardiac puncture), liver, heart and quadriceps were collected at 0.5, 1, 2, 4, 8 and 24 h after administration. Urine was collected during first 16 h from 24-h groups using metabolic collection cages. Concentrations of ASO and Palm-ASO were determined by LC–MS.

Mice homozygous for caveolae protein 1 (Cav1) knock-out on a C57BL/6J background (*Cav1^tm1Mls^*) were obtained from The Jackson Laboratory (Strain 007083) under license from Albert Einstein School of Medicine. Age and gender matched C57BL/6J mice were used as controls (18). We administered ASO or Palm-ASO to 8–12-week-old male and female (*n* = 4/test article/dose: 2 male + 2 female) mice at 1.3, 2.5, 5, 10 or 20 μmol/kg. An untreated control group of each strain was included (*n* = 4: 2 male + 2 female). The study was terminated 72 h after administration; plasma, liver, heart and quadriceps were collected; and MALAT1 RNA normalized to cyclophilin mRNA was determined in tissues by qRT-PCR.

Homozygous neonatal Fc receptor (FcRn) α-chain knock-out mice (*Fcgrt^tm1Dcr^*) first described by Roopenian *et al.* were obtained from The Jackson Laboratory (Strain 003982) and referred herein as FcRn^−/−^ mice (19). Age-matched C57BL/6J mice were used as controls. We treated 8–12-week-old, male FcRn^−/−^ mice and C57BL/6J mice (*n* = 4/test article/dose) with a single subcutaneous administration of ASO at 1, 3, 10 or 30 μmol/kg or Palm-ASO at 0.3, 1, 3 or 10 μmol/kg and determined MALAT1 RNA expression normalized to cyclophilin mRNA in liver, heart and quadriceps by qRT-PCR 72 h after dosing.

Homozygous albumin knock-out mice on a C57BL/6J background (*C57BL/6J-Alb^em8Mvw^/MvwJ*), first described by Roopenian *et al.* were obtained under license from The Jackson Laboratory (Strain 025200) (20). Age and gender-matched C57BL/6J mice were used as controls. Groups of six mice, three males and three females, of each strain, aged 6–16 weeks old were subcutaneously administered ASO at 0.07, 0.18, 0.74, 1.85, 7.4 or 18.5 μmol/kg; or Palm-ASO at 0.04, 0.09, 0.37, 0.92, 3.7 or 9.2 μmol/kg. An untreated group of six mice of each strain were used as controls. Mice received weekly administrations for 3 weeks. Study was terminated 96 h after the last administration when plasma, liver, heart and quadriceps were collected. MALAT1 RNA was quantified in tissues by qRT-PCR, normalized to cyclophilin mRNA, and reported relative to untreated controls. ED_50_ values were calculated as described later, except that the top of the three-parameter nonlinear fit used to determine the ED_50_ in quadriceps was constrained to a shared value of 1.303.

### Determination of RNA expression by qRT-PCR and determination of ED_50_

Reduction of MALAT1 RNA expression was determined by qRT-PCR using StepOne RT–PCR instruments (Applied Biosystems). RNA was extracted from tissues using PureLink Pro 96 Total RNA Purification Kit (Life Technologies, Carlsbad, CA, USA) with only slight modifications to the supplier's protocol. Briefly, 50–100 mg tissue was homogenized and lysed in 2 ml guanidine isothiocyanate with 2% β-mercaptoethanol. 50 ul lysate was diluted with 300 ul PureLink lysis buffer and then further diluted with 350 ul 70% ethanol. 700 ul diluted tissue lysate was then loaded onto PureLink filter plates and we followed the supplier's protocol including DNase digest through elution of RNA with 80 ul water. MALAT1 RNA and Cyclophilin A mRNA was measured by qRT-PCR using Express One-Step SuperMix qRT-PCR Kit (Life Technologies, Carlsbad, CA, USA). Primers and probes for the qRT-PCR analysis of MALAT1 and Cyclophilin A were obtained from Integrated DNA technologies (Coralville, IA, USA). MALAT1 primers consisted of a forward DNA primer TGGGTTAGAGAAGGCGTGTACTG; a reverse DNA primer TCAGCGGCAACTGGGAAA; and a DNA probe with 3′ TAMRA and 5′ fluorescein CGTTGGCACGACACCTTCAGGGAC. Mouse Cyclophilin A primers consisted of a forward DNA primer, TCGCCGCTTGCTGCA; a reverse DNA primer, ATCGGCCGTGATGTCGA; and a DNA probe with 3′ TAMRA and 5′ fluorescein, CCATGGTCAACCCCACCGTGTTC. The assay is based on a target-specific probe labeled with a fluorescent reporter and quencher dyes at opposite ends. The probe is hydrolyzed through the 5′-exonuclease activity of Taq DNA polymerase, leading to an increasing fluorescence emission of the reporter dye that can be detected during the reaction. Serial dilutions of RNA from untreated controls were used as standards. Cq values ranged from 17 to 26; efficiencies ranged from 95 to 120% and *R*^2^ values ranged from 0.985 to 0.998. Technical replicants of each sample were analyzed in triplicate. MALAT1 RNA levels were normalized to Cyclophilin A mRNA expression. ED_50_ values were determined with GraphPad Prism 7 software by plotting the log dose of ASO against normalized MALAT1 RNA expression relative to untreated controls. Unless otherwise noted, the data were fitted using a three-parameter nonlinear fit with variable slope and constraining the bottom to 0 and top to 1. ED_50_ values of ASO and Palm-ASO are reported along with 95% confidence intervals and *P*-values describing the comparison of the nonlinear fits using an extra sum-of-squares *F* test.

### Extraction and quantitation of ASO by LC–MS

ASO and Palm-ASO were extracted from 50 to 100 ul plasma or 100–200 mg tissues as previously described using phenol/chloroform followed by solid phase extraction of the aqueous extract using phenyl-functionalized silica sorbent (Biotage, Uppsala, Sweden) (21). Extracts were analyzed by LC/MS and concentrations determine using a method similar to that described by Gaus *et al.* (22) Briefly, separation was accomplished using an 1100 HPLC–MS system (Agilent Technologies, Santa Clara, CA, USA) consisting of a quaternary pump, UV detector, a column oven, an autosampler and a single quadrupole mass spectrometer. Samples were injected on an X-bridge OST C18 column (2.1 × 50 mm, 2.5-μm particles; Waters, Milford, MA, USA) equipped with a SecurityGuard C18 guard column (Phenomenex, Torrance, CA, USA). The columns were maintained at 55°C. Tributylammonium acetate buffer (5 mM) and acetonitrile were used as the mobile phase at a flow rate of 0.3 ml/min. Acetonitrile was increased as a gradient from 20% to 70% over 11 min. Mass measurements were made online using a single quadrupole mass spectrometer scanning 1000–2100 *m*/*z* in the negative ionization mode. Molecular masses were determined using the ChemStation analysis package (Agilent, Santa Clara, CA, USA). Peak areas from extracted ion chromatograms were determined for ASO and IS and a trendline established using the calibration standards, plotting concentration of ASO against the ratio of the peak areas ASO/IS. Concentration of ASO in study samples were determined using established trendlines and reported as μg ASO/g tissue.

### Lymph and plasma PK in rats

A previously described surgical model for lymph collection in rats was established at Hilltop Labs (Scottdale, PA, USA) (23). All studies were performed in accordance with the AAALAC guidelines and under IACUC-approved protocols. Male Sprague-Dawley rats (10-week-old, 315–355 g) were anesthetized with a combination of Dextomitor and Telazol and remained anesthetized throughout the surgery and study and were not permitted to recover. Rats were cannulated at the left thoracic duct and duodenum. Cannulated rats (*n* = 3/test article) and non-cannulated control rats (*n* = 3/test article) were administered ASO or Palm-ASO via tail vein injections. Lymph was collected continuously changing the collection vial at 11 different time periods starting with a collection 15 min pre-administration. 100 ul blood was collected on K2EDTA via tail snip at the end of each lymph collection period. Additionally, to reduce the risk of dehydration, sterile saline was infused as a bolus through the intraduodenal cannula at a volume equal to the lymph volume collected for each period. Following the final collection, rats were euthanized by asphyxiation with carbon dioxide. Plasma was separated from blood by centrifugation and all samples stored and shipped on dry ice to Ionis Pharmaceuticals for analysis. Plasma and lymph samples were extracted and analyzed by anion exchange chromatography.

### Quantitation of ASO in plasma and lymph by anion exchange chromatography (AEX)

AEX running buffer A, consisted of 20% ACN, 3 M urea, 1 mM EDTA and 25 mM Tris, pH 8; buffer B included 1 M NaClO_4_. Plasma and lymph samples were diluted directly in AEX buffer A. We prepared standards of ASO and Palm-ASO in control plasma and lymph at 1 μM and serial dilution in plasma or lymph to 3.9 nM. A PNA oligonucleotide with a sequence fully complimentary to ASO and labeled at the 5′ end with ALEXA488 was added at 2 uM to standards and samples and then heated to 80°C for 2 min and allowed to cool to room temperature for at least 30 min to allow the PNA and ASO or Palm-ASO to hybridize. Duplexed samples were separated on AEX column DNAPac PA200 4 × 250 nm (Thermo Scientific, Waltham, MA, USA) using an Agilent 1100 series LC. Samples were separated with a flow rate of 1 ml/min and a gradient running from 5% to 35% B over 10 min. An Agilent FLD G1321A detected fluorescence using Ex488/Em520. Under these conditions, PNA elutes at 1.2 min, PNA duplexed ASO elutes at 4.9 min, and PNA-duplexed Palm-ASO elutes at 5.6 min (all times ± 3%). Standard curves were established for both ASO and Palm-ASO in control plasma and lymph by plotting concentration against FLD peak area and unknown samples calculated from these standard curves. Technical replicants were run in duplicate.

### Fluorescence polarization

Binding of ASO and Palm-ASO to plasma proteins was determined using fluorescence polarization (FP) as described by Gaus *et al.* (24) Briefly, ASO and Palm ASO were labeled at the 3′ terminus with ALEXA647. Binding measurements were conducted in 1× DPBS (Gibco) in flat-bottom non-binding 96-well plates (Corning, NY, USA) at 25°C. ALEXA647-labeled ASO or Palm-ASO were added at a final concentration of 2 nM to solutions of protein ranging from sub nM to mM concentrations. Solutions equilibrated at least 30 min before measuring fluorescence polarization (λ_ex_ = 635 nm, λ_em_ = 675 nm) on a Tecan InfiniteM1000 Pro (Baldwin Park, CA, USA). Using polarized excitation and emission filters, the instrument measures fluorescence perpendicular (P) to the excitation plane (the ‘P-channel’) and fluorescence that is parallel (S) to the excitation plane (the ‘S-channel’). FP is calculated in millipolarization units (mP) as follows: mP = [(S – P * G)/(S + P * G)] × 1000, where G (‘G-factor’) is measured by the instrument as a correction for any bias toward the P channel (25). Polarization values of each ALEXA647-labeled ASO in DPBS at 2 nM concentration were subtracted from each measurement. *K*_d_ values were calculated with GraphPad Prism 7 software (GraphPad Software, La Jolla, CA, USA) using non-linear regression for curve fit assuming one binding site.

### *In vitro* activity

HepG2 cells were plated on collagen-coated 96-well plates (3000 cells/well) in complete medium: MEM (Gibco) supplemented with 10% FBS and penicillin/streptomycin. After 24 h, media was replaced with serum-free MEM. After another 24 h, media was replaced with complete media; serum-free media; media with 2%, 5% or 10% FBS; or serum-free media with 0.1% BSA, 0.2% BSA or 0.4% BSA. At the same time, ASO, Palm-ASO or Control-ASO was added to the cells at a final concentration of 2 or 10 μM. Treatments were performed in triplicate. Cells were lysed after 24 h and RNA extracted using PureLink Pro 96 Total RNA Purification Kit according to supplier's protocol. MALAT1 RNA and actin mRNA were quantified by qRT-PCR as described above with StepOne RT-PCR instruments. MALAT1 was quantified using a forward DNA primer GAATTGCGTCATTTAAAGCCTAGTT, a reverse DNA primer TCATCCTACCACTCCCAATTAATCT and a DNA probe with 3′ TAMRA and 5′ fluorescein, ACGCATTTACTAAACGCAGACGAAAATGGA. Actin was quantified using a forward DNA primer ATTGCCGACAGGATGCAGAA, a reverse DNA primer GCTGATCCACATCTGCTGGAA, and a DNA probe with 3′ TAMRA and 5′ fluorescein, CAAGATCATTGCTCCTCCTGAGCGCA. PCR was run with triplicate technical replicates. MALAT1 RNA was normalized to actin mRNA and MALAT1 expression reported relative to untreated cells cultured in complete media. Knockdown is reported as average percent knockdown from untreated cells or average percent expression relative to untreated cells along with standard deviation and p-values determined by ordinary two-way ANOVA and Tukey's multiple comparison test.

### Proteomic analysis of mouse plasmas

Aliquots of plasma collected on K2-EDTA were sent to MRM Proteomics (Montreal, QC, Canada) and analyzed with their Peptiquant Proteomic Assay for mouse plasma.

## RESULTS

### Conjugation of ASO with palmitic acid increases activity in heart and quadriceps

Conjugation of ASO with palmitic acid enhanced potency 3–4-fold in heart and quadriceps compared with the unconjugated ASO (Figure 1). ED_50_ of ASO and Palm-ASO in heart were 6.7 and 2.2 μmol/kg respectively and nonlinear regressions were statistically different (*P* = 0.0009). ED_50_ of ASO and Palm-ASO in quadriceps were 8.3 and 2.2 μmol/kg respectively and were statistically different (*P* < 0.0001). ED_50_ of ASO and Palm-ASO in liver were 1.0 and 0.5 μmol/kg respectively and were statistically different (*P* < 0.0001).

### Early distribution of palmitic acid conjugated ASO *in vivo*

We hypothesized that increased affinity for plasma proteins, albumin in particular, would increase Palm-ASO plasma and tissue exposure compared with ASO. To determine the effect of palmitic acid conjugation on ASO distribution, we administered 7.4 μmol/kg (40 mg/kg ASO) ASO or Palm-ASO subcutaneously to mice and collected plasma, liver, quadriceps and heart 0.5–24 h after administration. All tissues had greater exposure to Palm-ASO compared with ASO in the first 24 h after administration as measured by AUC (Figure 2A). AUC of Palm-ASO in plasma was 8-fold greater than ASO: 896 ± 159 compared with 108 ± 16 μg/ml·min respectively. AUC of Palm-ASO in liver was nearly 2-fold greater than ASO: 1971 ± 180 compared with 1128 ± 44 μg/ml·min. AUC of Palm-ASO in heart was 2.5-fold greater than ASO: 310 ± 22 compared with 126 ± 8 μg/ml·min. AUC of Palm-ASO in quadriceps was only slightly greater, however, statistically significant, than ASO: 113 ± 4 compared with 80 ± 12 μg/ml·min. Significantly more ASO was found in urine when mice were dosed with ASO compared with Palm-ASO: 218 μg, or 18% of the total 40 mg/kg dose compared with 3.9 μg or 0.3% of the dose respectively. Thus, conjugation of ASO with palmitic acid clearly results in less excretion of ASO in urine and greater exposure of multiple tissues to ASO.

Histological analysis of tissue samples immunostained for PS ASO concurred with PK analysis and demonstrated greater accumulation of ASO in tissues of mice dosed with Palm-ASO compared with ASO. Palm-ASO staining at early time points was diffuse and localized primarily along inter-cellular boundaries suggesting that the majority of the Palm-ASO was limited to the interstitial space or plasma membrane, with only a very small increase in intracellular ASO. Figure 2B shows immunostaining of quadricep tissue at 0.5, 1, 2, 4, 8 and 24 h. At 1 h, there is clearly greater staining for ASO in tissue from a mouse administered Palm-ASO compared with the mouse dosed with ASO, but the staining is nearly entirely extracellular; by 4 h, there is some punctate staining suggesting intracellular localization of ASO; and by 8–24 h, much of the ASO is cleared from the tissue with some punctate intracellular staining. This rapid accumulation and clearance from tissue suggests that palmitic acid conjugation facilitates passage of ASO from circulation to the interstitium of tissues, however, Palm-ASO is cleared from tissue before significant concentrations are taken up intracellularly.

### Early distribution to lymphatics

To better understand the accumulation and clearance of Palm-ASO from tissues, we evaluated the early distribution of ASO and Palm-ASO in plasma and lymph of rats after intravenous administration. After distribution of ASO from circulation to tissues, it is cleared from tissues and returned to circulation through the lymphatic system. The lymphatic system provides a unidirectional route for movement of interstitial fluid from tissues into cardiovascular circulation. About 75% of lymph flows through the thoracic lymph duct and drains into circulating blood at the brachiocephalic vein (26). We sampled fluids returning to blood at the thoracic lymph duct to determine the rate of ASO clearance from tissue through this route and metabolic state of Palm-ASO cleared through this rout (i.e. is Palm-ASO cleared in lymph intact or is the palmitate moiety cleaved from the ASO).

**Figure 3.**
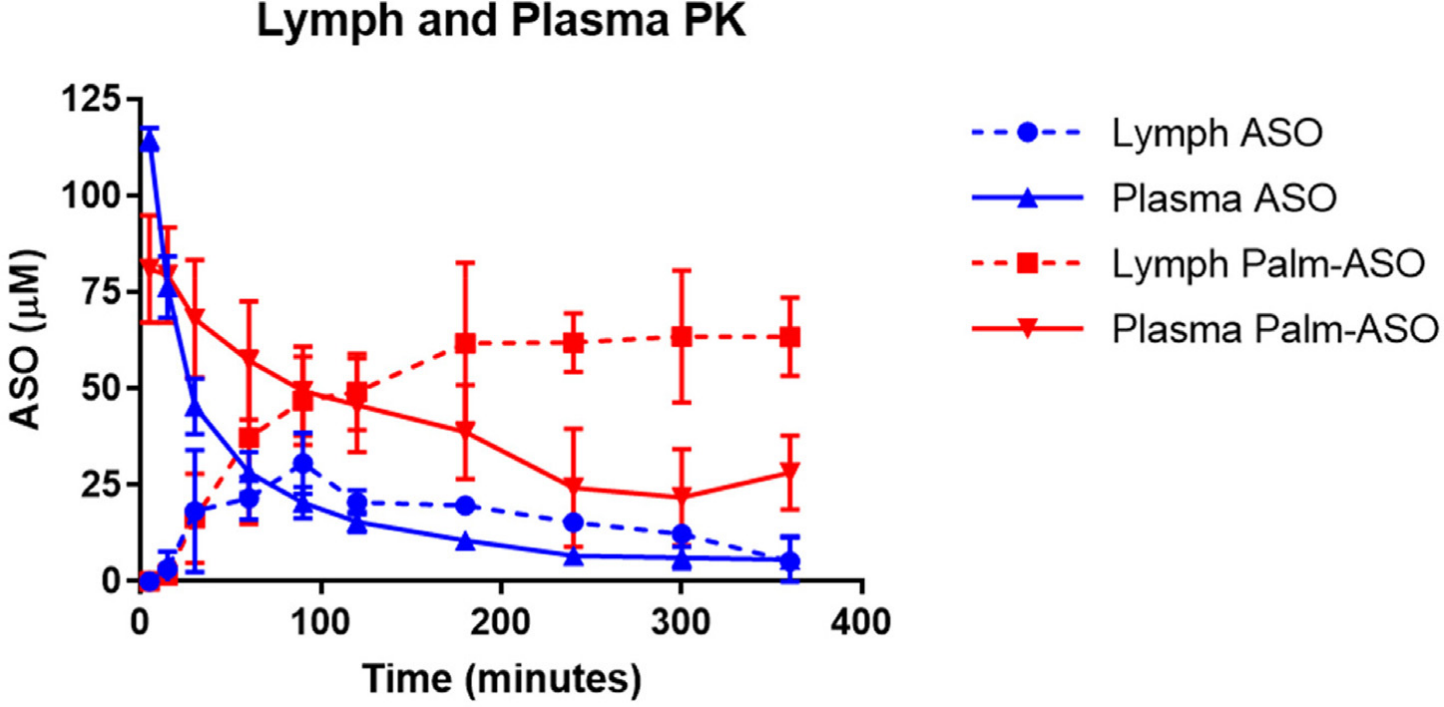
AUC of Palm-ASO is significantly increased for Palm-ASO in both plasma and lymph compared with ASO. Rats were administered 7.5 μmol/kg ASO or Palm-ASO via tail vein injection. Plasma was collected by tail snips at various time points from 5 to 360 min. Lymph was collected via a cannulated thoracic lymph duct at the same time points.

As described in the methods section, we administered 7.4 μmol/kg to Sprague Dawley rats cannulated at the thoracic lymph duct and collected plasma and lymph samples starting 15 min before administration through 360 min after administration. ASO and Palm-ASO concentrations in plasma and lymph were quantified by anion exchange chromatography (AEX) (Figure 3). While the duration of this experimental model is not adequate to observe steady-state pharmacokinetics, we can observe the early plasma and lymph kinetics. As we observed in mice, plasma AUC of Palm-ASO was >2-fold greater than ASO in rats (±SD): 14.1 ± 1.2 compared with 6.3 ± 0.3 mM·min. Lymph AUC of Palm-ASO was >3-fold greater than ASO (±SD): 18.3 ± 1.4 compared with 6.0 ± 0.4 mM·min. ASO concentrations increased in lymph over the first 90 min, at which time, concentrations declined at a rate similar to plasma clearance. In contrast, Palm-ASO accumulated in lymph over 180 min to concentrations well above plasma concentrations, at which time Palm-ASO concentrations remained steady for the duration of the 360-min study. Importantly, nearly all Palm-ASO collected in lymph was found intact. That is, <2% of ASO collected in lymph of animals dosed with Palm-ASO was not conjugated, indicating that metabolism of Palm-ASO in the plasma, interstitium or lymph is minimal.

### Affinity of lipid conjugated ASO for albumin, HDL and LDL

**Figure 4.**
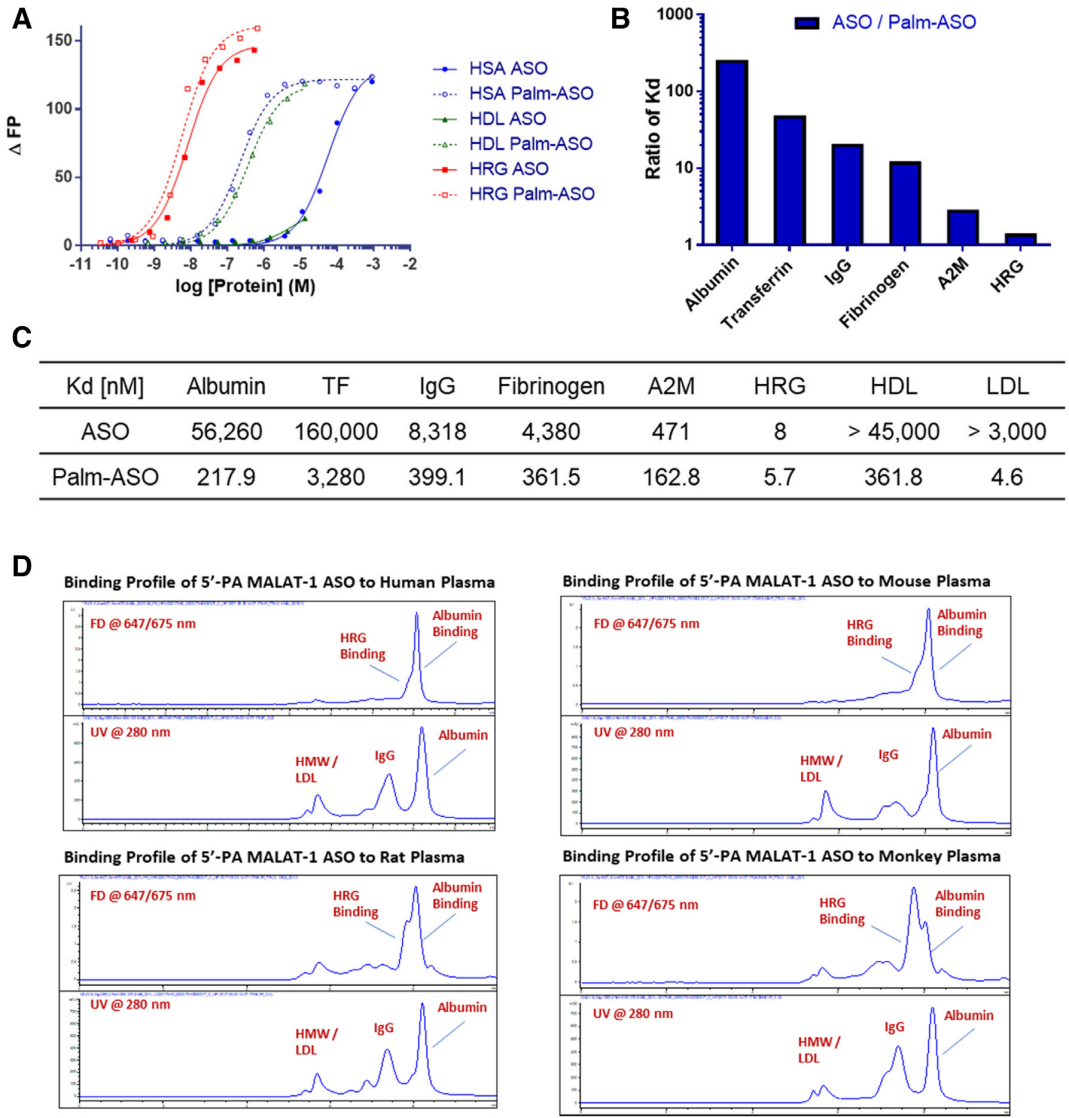
Palmitate conjugation increases ASO affinity for several plasma proteins and lipid complexes. Binding of ASO and Palm-ASO to a set of human plasma proteins: albumin, transferrin (TF), IgG, fibrinogen, α-2-macroglobulin (A2M) and histidine-rich glycoprotein (HRG); and lipid particles: HDL and LDL. (**A**) FP binding curves for human serum albumin, HDL and HRG. (**B**) Ratio of *K*_ds_ ASO/ Palm-ASO. (**C**) Table of *K*_ds_. (**D**) ASO and Palm-ASO binding profiles in human and mouse plasma by SEC.

We determined the affinities of ASO and Palm-ASO using fluorescence polarization (FP) for several plasma proteins including albumin, transferrin (TF), IgG, fibrinogen, alpha-2-macroglobulin (A2M), histidine rich glycoprotein (HRG), and isolated high-density lipoproteins (HDL) and low-density lipoproteins (LDL) (Figure 4A). Except for HRG, which exhibits low nM affinity for both ASO and Palm-ASO, Palm-ASO demonstrated between 2-fold and >200-fold greater affinity for all proteins evaluated (Figure 4B and C). Notably, the affinity for albumin, by far the most abundant plasma protein, increased 200-fold with conjugation of palmitate from 56 μM for ASO to 218 nM for Palm-ASO. ASO has relatively low affinity for the lipid particles and precise KDs could not be determined by FP. However, Palm-ASO exhibited relatively high affinity for HDL and LDL with 319 and 4 nM KD respectively. While Palm-ASO exhibited high affinity for isolated lipid particles in the FP assay, size exclusion chromatography of fluorescently-labeled Palm-ASO in plasma demonstrated very little binding to high molecular weight components like lipid particles, but rather binds primarily to components of plasma with molecular weights in the 60–80 kDa range, likely albumin and HRG (Figure 4D).

### Palm-ASO activity in Cav^−/−^ mice

**Figure 5.**
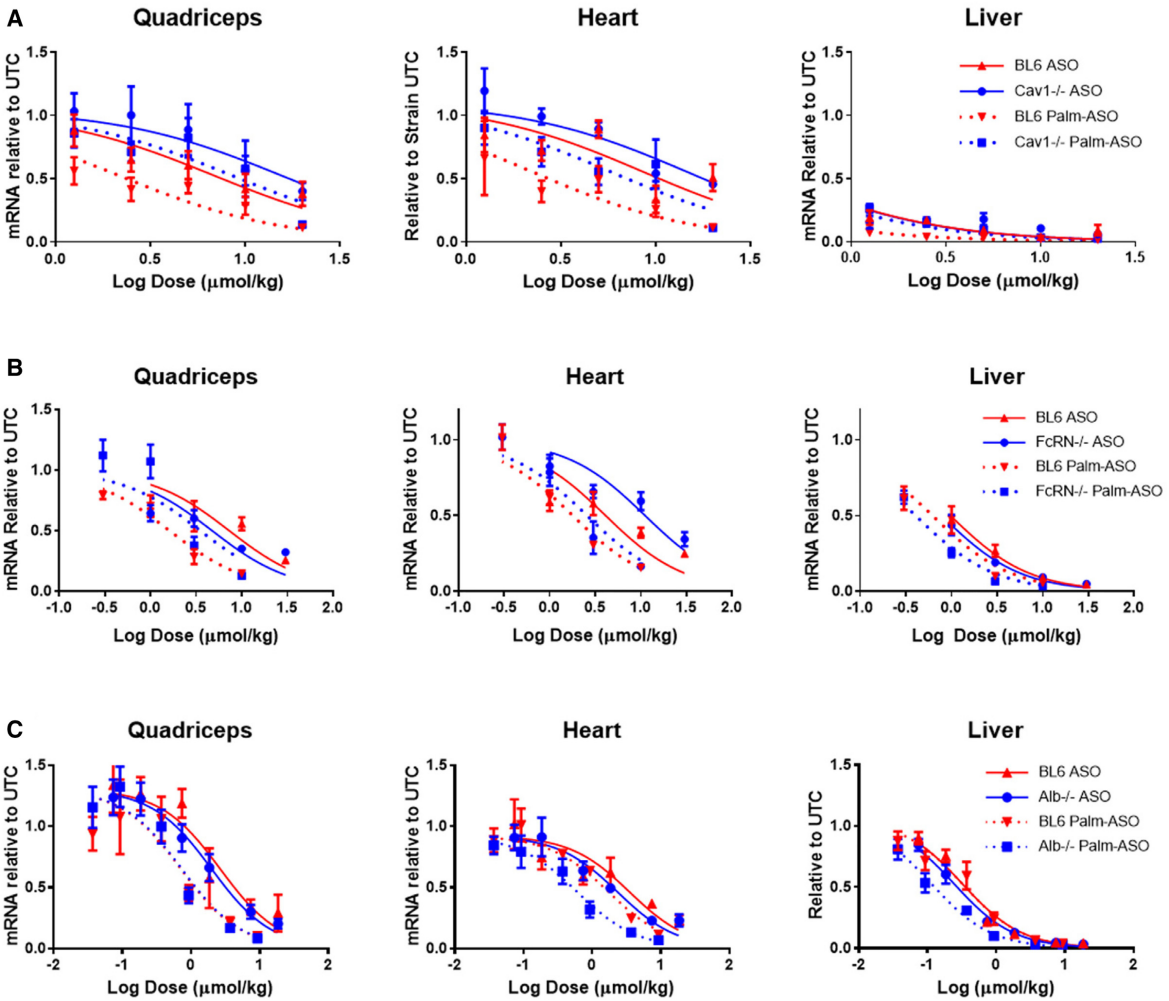
Activity of ASO and Palm-ASO is attenuated in Cav1^−/−^ and FcRn^−/-^ mice, but not Alb^−/−^ mice. (**A**) Activity of both ASO and Palm-ASO is attenuated in Cav1^−/−^ mice. The ED50 for both ASO is shifted in the Cav1^−/−^ mice with a more pronounced effect on Palm-ASO. This suggests both ASO are trafficked, at least in part, through Caveolin-1 dependent pathways. (**B**) Activity of ASO and Palm-ASO is attenuated in FcRn^−/−^ mice, with a more pronounced effect on Palm-ASO. (**C**) Activities of both Palm-ASO and ASO are unchanged in quadriceps of Alb^−/−^ mice compared to BL6 and slightly potentiated in heart and liver.

To investigate the role of caveolin-1 (Cav1) in the activity of ASO, we compared activities of ASO and Palm-ASO in wild type C57Bl/6 mice with caveolin-1 knockout (Cav1^−/−^) (Figure 5A). Both ASO and Palm-ASO were less active in quadricep and heart of Cav^−/−^ mice compared to wild type, with Palm-ASO attenuated to a greater extent than ASO. Consistent with previous experiments in wild-type mice, we observed ∼60% lower ED_50_ in quadricep for Palm-ASO (2.4 μmol/kg) compared with ASO (7.9 μmol/kg). In Cav^−/−^ mice, the ED_50_ of ASO in quadricep increased 2-fold compared in wild-type mice (17.9 μmol/kg), while ED_50_ of Palm-ASO increased 4-fold (9.7 μmol/kg) compared to wild-type. Likewise, both ASO and Palm-ASO have reduced activity in Cav^−/−^ mice compared to wild type with Palm-ASO affected more than ASO. This attenuation of activity suggests that Cav1 may play an important role in productive transport of ASO and Palm-ASO to muscle tissue. Activity in the liver appeared similar across mouse strains, however, the doses used were designed to observe knockdown in muscle and were too high to compare activity in liver.

### Palm-ASO activity in FcRn^−/−^ mice

Testing our hypothesis that increased activity of Palm-ASO compared with ASO is related to increased affinity and trafficking with albumin, we examined the activity of Palm-ASO in FcRn**^−^^/–^** mice. Both IgG and albumin have reduced circulation half lives in FcRn**^−^^/^^−^** mice compared with wild type. These mice have 45% reduction in circulating albumin due to loss of FcRn-mediated recycling and drastically reduced transfer of IgG from mother to neonate due to loss of FcRn-mediated transcytosis (19,27,28).

With faster clearance of albumin from circulation and reduced endothelial transcytosis of albumin, we expected attenuation of Palm-ASO activity in this mouse model. Indeed, while Palm-ASO exhibited >4-fold greater activity compared to ASO in quadriceps of C57BL/6J mice (1.6 μmol/kg compared with 7.3 μmol/kg, respectively *P* < 0.0001), the activity of Palm-ASO was attenuated in quadriceps of FcRn^−/−^ mice compared with C57BL/6J (3.5 μmol/kg compared with 1.6 μmol/kg, *P* = 0.058) (Figure 5B). Furthermore, Palm-ASO did not exhibit significantly better activity than ASO in FcRn^−/−^ mice: Palm-ASO ED_50_ of 3.5 μmol/kg compared with ASO ED_50_ of 4.3 μmol/kg, *P* = 0.51. This suggests that FcRn may be involved in mediating the improved activity of Palm-ASO observed in quadriceps compared to ASO, perhaps through a putative role in transcytosis of albumin across vascular endothelium.

In contrast to quadriceps, Palm-ASO exhibited slightly better activity in liver of FcRn^−/−^ compared with C57BL/6J mice: ED_50_ of 0.40 μmol/kg and 0.62 μmol/kg respectively, *P* = 0.016. Activity of ASO was not significantly different between FcRn^−/−^ and C57BL/6J mice: 0.78 and 0.97 μmol/kg respectively, *P* = 0.22.

### Palm-ASO activity in Alb^−/−^ mice

We used a mouse model deficient in albumin to investigate the role of albumin trafficking in the improved activity of Palm-ASO compared to ASO (20). We hypothesized that the activity of both ASO and Palm-ASO would be reduced in the absence of albumin, a major carrier of ASO and Palm-ASO.

We administered Palm-ASO or ASO to wild-type C57BL/6 mice or Albumin (Alb^−/−^) deficient strain subcutaneously once a week for three weeks and collected tissues 3 days after the last administration to determine target RNA knockdown. Consistent with previous studies, we observed increased activity of Palm-ASO compared to ASO in heart and quadricep muscle of the wild-type mice (Figure 5C). The activities of both ASO and Palm-ASO in quadriceps were similar in quadriceps of Alb^−/−^ mice compared to wildtype. ED_50_ of Palm-ASO was 0.73 μmol/kg in quadriceps of Alb^−/−^ and 0.71 μmol/ml in quadriceps of BL6; ED_50_ of ASO was 2.0 μmol/kg in quadriceps of Alb^−/−^ and 2.7 μmol/kg in BL6. Likewise, ASO activity remained relatively unchanged in heart and liver of Alb^−/−^ mice compared with wildtype, while Palm-ASO was slightly more active in Alb^−/−^ mice. This contrasted with the Cav^−/−^ and FcRn^−/−^ models where both ASO and Palm-ASO lost activity.

To better understand the plasma proteome of Alb^−/−^ mice, we use mass spectrometry proteomics to identify differential expression of plasma proteins in Alb^−/−^ mice compared to wildtype. We found that nearly all plasma proteins were approximately doubled in concentration in the Alb^−/−^ mice compared to wildtype mice. There was no overexpression of a single protein to fully compensate for the reduced concentration of albumin, We included samples from the other mouse models discuss here, Cav1^−/−^ and FcRn^−/−^ mice, and other than finding ∼40% less abundant albumin in the FcRn^−/−^ model, there were no remarkable changes in the plasma proteomes (Supplementary Figure S1 and Supplementary Table S1).

### Effect of plasma proteins on *in vitro* activity of lipid conjugated ASO

**Figure 6.**
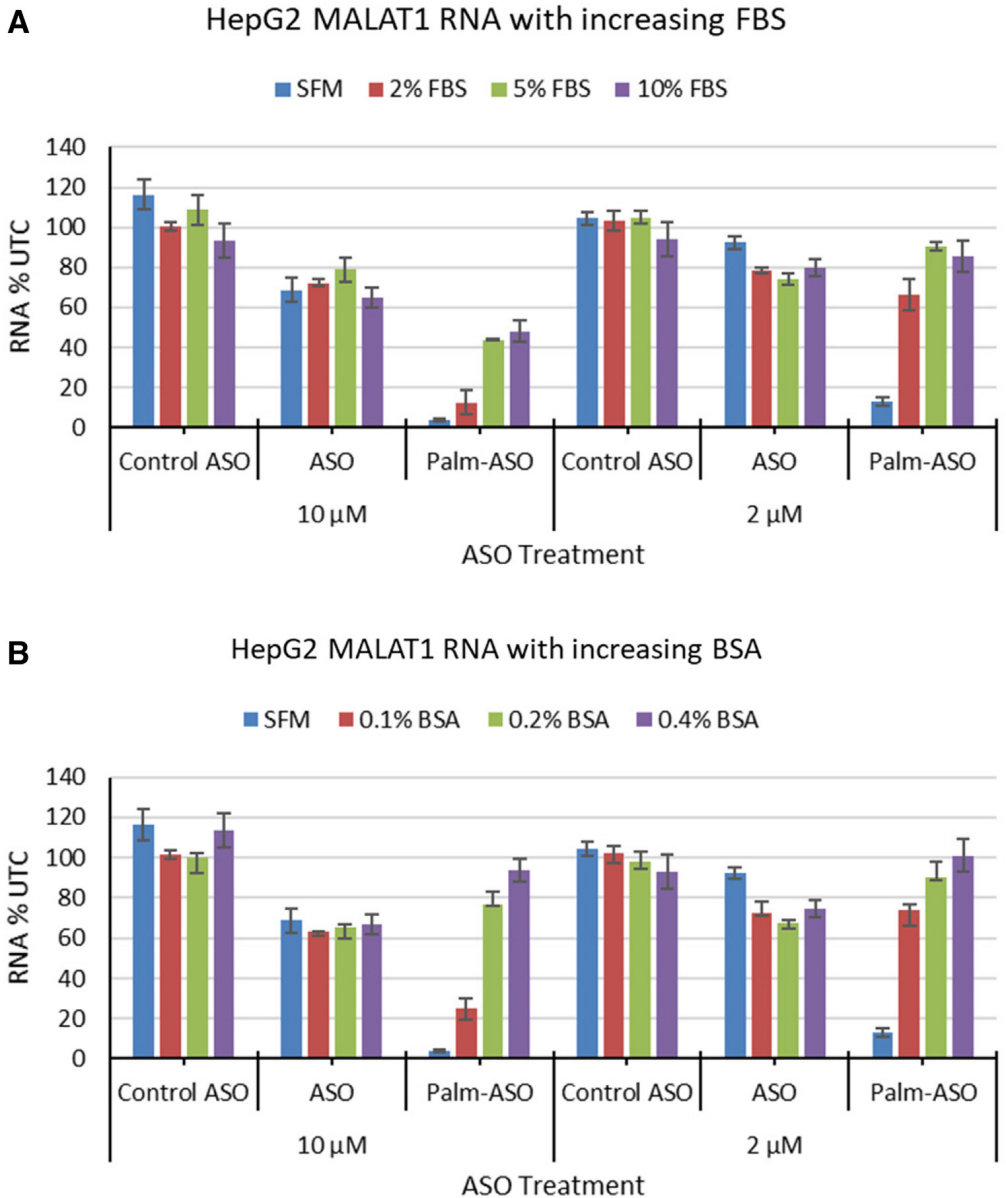
Increased serum or albumin decreases *in vitro* activity of Palm- ASO. (**A**) HepG2 cells were treated with 2 or 10 μM ASO or Palm-ASO for 24 h in complete media (10% FBS), 5% FBS, 2% FBS or serum-free media (SFM). ASO activity was unaffected by changes in FBS in the media while Palm-ASO activity was dramatically increased with lower serum levels. (**B**) Similarly, supplementing SFM with increasing concentrations of BSA reduces activity of Palm-ASO but has no effect on the activity of ASO in HepG2 cells.

To further investigate the correlation between plasma protein binding of ASO and ASO activity, we examined the effect of serum proteins on *in vitro* activity of ASO and Palm-ASO. Hep G2 were cultured in complete media and then treated with ASO or Palm-ASO targeting MALAT1 at 2 and 10 μM in complete medium with 10% FBS, 5% FBS, 2% FBS or serum-free medium (Figure 6A). When treated in media with 10% FBS, Palm-ASO induces only slightly more knockdown at 10 μM than ASO: 52 ± 5.5% knockdown compared with 36 ± 4.9% knockdown of MALAT1, *P* = 0.0024. However, in serum-free media, treatment with 10 μM Palm-ASO results in 96 ± 0.5% knockdown, significantly greater than ASO activity with 31 ± 6% knockdown, *P* < 0.0001. While ASO activity at 2 or 10 μM is unaffected by serum concentration, Palm-ASO is attenuated by FBS in a concentration-dependent manner.

We observed a similar effect when serum-free media was supplemented with increasing concentrations of bovine serum albumin. No significant change was observed in ASO activity in 0.1% BSA (37 ± 6% knockdown) compared to 0.4% BSA (33 ± 5% knockdown), *P* = 0.8. However, 10 μM Palm-ASO was inactive in 0.4% BSA (6 ± 5% knockdown), achieved 23 ± 6% knockdown in 0.2% BSA (*P* = 0.0016 compared to 0.4% BSA), and achieved 75% ± 5% knockdown in 0.1% BSA (*P* < 0.0001 compared with 0.2% BSA) (Figure 6B). Together these data suggest, along with our observations that Palm-ASO has higher affinity for a variety of proteins compared with ASO, that strong binding to plasma proteins, including albumin, can inhibit productive uptake in cells. This is consistent with what has been reported with cholesterol-conjugated siRNA, where removal of serum enhanced activity in culture. (29)

## DISCUSSION

Conjugation of antisense oligonucleotides with palmitic acid has been demonstrated to improve activity in extrahepatic tissues (9,10). We hypothesized that this increase in activity is related to increased plasma circulation and increased exposure of extrahepatic tissue to ASO. We found the AUC of Palm-ASO was greater than ASO by 400% in plasma, 100% in liver, 50% in quadriceps and 250% in heart. While approximately 18% of the 40 mg/kg ASO dose was excreted in the urine over 24 h, only 0.3% of the same dose of Palm-ASO is excreted in the urine over the same period indicating a substantially greater portion of Palm-ASO at this dose is retained and distributed systemically compared to naked ASO. Histology staining for ASO in sections of heart and quadriceps collected at early time points, 0.5–4 h, suggest that Palm-ASO is primarily localized to the interstitial space at these early time points where the largest increase in tissue concentration is observed. This interstitial accumulation peaks around 2–4 h and is then cleared from these muscle tissues. Palm-ASO is not observed intracellularly in these tissues until 8–24 h after administration. It appears that naked ASO follows this same pattern of early tissue accumulation and then clearance over 24 h, but Palm-ASO concentrations are much higher. Thus, conjugation of ASO with palmitic acid exposes muscle tissues to greater concentrations of ASO within a couple hours of administration, but ASO is not taken up intracellularly in this time frame and the bulk of ASO is cleared from tissue without being distributed to myocytes.

To better characterize and determine if Palm-ASO is cleared from the interstitium as an intact conjugate or if it is metabolized in tissue before being cleared, we considered lymph as a proxy for interstitial fluid and implemented a surgical rat model in which the thoracic lymph duct is cannulated to allow continuous collection of lymph. We collected plasma and lymph up to 6 h after tail vein administration of Palm-ASO or ASO and determined ASO concentrations and analyzed by LC/MS to determine whether the ASO was conjugated in effluent lymph. All ASO collected in the lymph of rats administered Palm-ASO remained conjugated, indicating that Palm-ASO is cleared intact and not metabolized in the plasma, interstitium or lymph. As observed in mice, rat plasma AUC of Palm-ASO is increased ∼2-fold over ASO. When administered naked ASO, ASO accumulates in lymph as it is cleared from plasma until ∼100 min, at which point lymph concentration equals the concentration in plasma and ASO concentrations in both compartments decrease in parallel. In contrast, as Palm-ASO is cleared from plasma, concentrations in lymph increase and concentrations of the two compartments are equal at ∼100 min, however, lymph concentrations continue to increase as plasma concentrations continue to fall and concentrations plateau from ∼200 min out to the latest time point collected, 360 min. This is consistent with PK and histological evidence that not only does palmitic acid conjugation deliver greater concentrations of ASO to the interstitial space but retains ASO in the interstitium and lymphatics longer. Despite the greater exposure to tissues, however, Palm-ASO does not appear to efficiently distribute from the interstitium to myocytes and thus this increased exposure does not translate to a proportional increase in intracellular concentration and ASO activity; rather, these effects are less significant.

We hypothesized that the increased plasma and tissue exposure is mediated by increased affinity for albumin and increased transport along with albumin across continuous endothelium found in muscle tissue. Fluorescent polarization assays demonstrate a 200-fold greater affinity of Palm-ASO for albumin compared to ASO. While fluorescence polarization assays demonstrated palmitate conjugation greatly increases the affinity for several different plasma proteins and lipid particles, size exclusion chromatography suggests fluorescently labeled Palm-ASO primarily associates with plasma proteins ∼60–80 kDa, consistent with increased association with albumin.

To evaluate if this increased association with albumin mediates the increased activity in skeletal and cardiac muscle that we observe for Palm-ASO compared with ASO, we employed three different mouse models, each deficient in a component of albumin transport: caveolin-1 knockout mice (Cav1^−/−^), neonatal Fc receptor knockout mice (FcRn^−/−^) and albumin knockout mice (Alb^−/−^).

Caveolae-dependent transport represents a major pathway for endocytosis and transcytosis of albumin in most tissues, including continuous endothelium, with reports that as much as 65% of albumin transport across vascular endothelium may be caveolae-dependent (14,15,30,31). Caveolae are bulb-shaped invaginations of the plasma membrane defined by integral proteins known as caveolins and are especially abundant in endothelial tissues. Caveolin-1 (Cav1) is a 22 kDa protein expressed in many tissues with the notable exception of striated muscle and is essential for the formation of caveolae. Caveolin-1 knockout mice (Cav1^−/−^) were developed using targeted gene disruption and are viable and fertile despite lacking caveolae organelles in the endothelium and other tissues where Cav1 is expressed (32). These mice are deficient in endothelial uptake and transcytosis of albumin from vascular lumen to interstitium. However, Cav1 is an inhibitor of eNOS and the microvasculature, but not larger aortic vessels, of Cav1^−/−^ mice is rendered hyperpermeable to paracellular transit of radiolabeled-BSA as a result of increased nitric oxide (33,34). Thus, while the endothelium of Cav1^−/−^ mice lack caveolae and show major defects in uptake and transcellular movement of albumin, they demonstrate increased clearance of albumin from vascular circulation through increased paracellular transit. Interestingly, this increase in paracellular transit was not observed in studies using 5 nm gold-labeled BSA to track cellular uptake and transcytosis and the authors speculate that conjugation with 5-nm colloidal gold significantly alters the conformation and/or size of BSA, such that it can no longer pass through tight junctions. Although we do not address this here, it is likely that albumin or other large carrier proteins bound to a 16-mer ASO or Palm-ASO would also be precluded from paracellular transport. The activity of both ASO and Palm-ASO were attenuated in quadriceps and heart muscle of Cav1^−/−^ mice compared to wild type suggesting that both conjugated and unconjugated ASO are at least in part transported through Cav1 dependent pathways. The potentiated activity we observed with Palm-ASO compared to ASO in wild type mice was diminished in this model, suggesting Cav1-dependent transport plays a role in mediating this potentiation.

Originally identified in the intestines of newborn rats as a receptor involved in transport of IgG from mothers’ milk, FcRn is expressed across species and tissues (35). In newborn rats, IgG is internalized by enterocytes through fluid-phase endocytosis or apical surface FcRn (36). The acidic pH of the gut lumen enables binding of FcRn to IgG and the FcRn-IgG complex is transcytosed to the basolateral side, where more neutral pH triggers release of IgG into circulation. While the role of FcRn in transcytotic processes is best characterized in the context of IgG trafficking, FcRn also binds albumin in a pH sensitive manner at a binding site independent of IgG. An important role of FcRn in recycling IgG and albumin has been well characterized and is the basis for the long half-lives of these proteins. As IgG and albumin are endocytosed though pinocytosis, these endocytic vesicles fuse with acidified vesicles containing FcRn. At low vesicular pH, FcRn binds IgG and albumin at different binding sites which results in sorting of FcRn complexes to recycling vesicles that return to the surface where exposure to more neutral pH triggers release of IgG or albumin from FcRn. Alternatively, proteins not complexed with FcRn are sorted to lysosomes and degraded. FcRn is expressed in specialized tissues including, enterocytes and goblet cells of large and small intestines, hepatocytes and Kupffer cells of the liver, and macrophages and antigen presenting cells, and relatively high expression of FcRn localized to vascular endothelial cells can be found in most tissues. With the FcRn^−/−^ mouse model, we observed a reduction in Palm-ASO activity and very little change in ASO activity in quadriceps and heart compared with wild type controls. The potentiation in activity we observed with Palm-ASO compared with ASO in wild type mice is diminished in heart and eliminated in quadriceps of FcRn^−/−^ mice. These data suggest that FcRn is involved in transport of both ASO and Palm-ASO and that transport by FcRn contributes to the improved ASO activity observed with palmitate conjugation. The improved activity of Palm-ASO in liver of FcRn^−/−^ mice may relate to the function of hepatic FcRn in returning albumin to circulation (37). In the absence of FcRn, Palm-ASO associated with albumin may be directed to endocytic pathways that result in increased activity in hepatocytes.

Albumin is the most abundant protein in blood, with typical concentrations ranging from 30 to 50 mg/ml in humans and 20 to 30 mg/ml in mice. In humans, albumin exhibits an exceptionally long half-life of ∼20 days, primarily as a consequence of its affinity for FcRn which rescues albumin from catabolic degradation. It is the primary regulator of osmotic pressure and serves as a carrier for many biomolecules including fatty acids, hormones, minerals and heavy metals, several classes of small molecule drugs, and therapeutic antisense oligonucleotides (24,38). An albumin-deficient strain of mice was recently developed using TALEN-mediated disruption of the albumin gene (20). The mice are analbuminaemic but healthy. We hypothesized that without albumin as a carrier, both ASO and Palm-ASO would lose activity in these mice and the difference in activity between ASO and Palm-ASO would be attenuated.

Somewhat surprisingly, we observed no change in ASO activity in heart, quadriceps, or liver of Alb^−/−^ mice compared to wild type controls. In contrast to Cav^−/−^ and FcRn^−/−^ mice, Palm-ASO activity was unchanged in quadriceps and slightly increased in activity in the Alb^−/−^ mouse model. To better understand these findings, we examined protein binding of ASO and Palm-ASO in plasma from Alb^−/−^ mice using size exclusion chromatography and fluorescence polarization. We found that despite lacking albumin, ASO and Palm-ASO primarily associate with proteins of about 60–80kDa and the affinity for plasma is not significantly different from plasma of wild-type mice. Looking to identify a compensatory protein that might be upregulated to reprise one or more roles of albumin in this knockout model, we found that no single protein was upregulated to the extent that it approached concentrations of albumin in wild-type mice. Rather, nearly all proteins roughly doubled in concentration while maintaining the same relative rank order in abundance. This is consistent with our observation that although albumin comprises roughly 60% of plasma protein in wild-type mice, Alb^−/−^ mice only have about 40% lower total protein. However, these data are confounding in that they suggest that despite an abundance of evidence to the contrary, actually albumin plays little or no role in transport of ASO and Palm-ASO or, more likely, these Alb^−/−^ mice survive only because a great number of proteins are upregulated to compensate for lost functions of albumin and some of these proteins have sufficient affinity for ASO and Palm-ASO that their activities remain relatively unchanged.

With the aim of increasing activity of ASO in skeletal and heart muscle, it was disappointing that while palmitic acid conjugation increased tissue exposure, likely through increased trafficking across continuous endothelium, the Palm-ASO failed to deposit in the parenchymal cells before being cleared from tissue. It may be that increased affinity for carrier molecules like albumin facilitates the increased plasma and tissue exposure and reduces excretion in urine but without a mechanism for disassociation from carrier proteins in the tissues, this increased affinity also prevents deposit or transfer of ASO to the parenchymal cells; the result being that the increase in ASO activity is only a fraction of the increased exposure. Indeed, we have evidence that plasma protein binding of ASO can interfere or inhibit cellular uptake of ASO *in vitro* (39). Increased serum protein concentration in culture media had little effect on ASO activity in HepG2 cells but significantly inhibited activity of Palm-ASO, likely by sequestering the Palm-ASO in the media and preventing uptake by the cells.

Conjugation of a Malat1-targeting ASO with palmitic acid increases activity of ASO in heart and skeletal muscle by ∼3–4-fold. We demonstrate that conjugation with palmitic acid increases affinity for albumin and other plasma proteins and lipid particles and propose that increased activity of Palm-ASO is mediated by increased affinity for carrier proteins, primarily albumin, which results in increased plasma and tissue exposure to ASO. In support of this hypothesis, we demonstrate that conjugation with palmitate significantly increase plasma and tissue AUC during the first 24 hours after subcutaneous administration. Furthermore, we demonstrate that the effect of palmitic acid conjugation on ASO activity is attenuated in mouse models deficient in albumin trafficking. While increasing affinity for plasma proteins increases plasma and tissue exposure, in vivo and *in vitro* data suggests greater affinity of conjugated ASO for serum proteins also inhibits uptake and activity of ASO in parenchymal cells.

**Figure 7.**
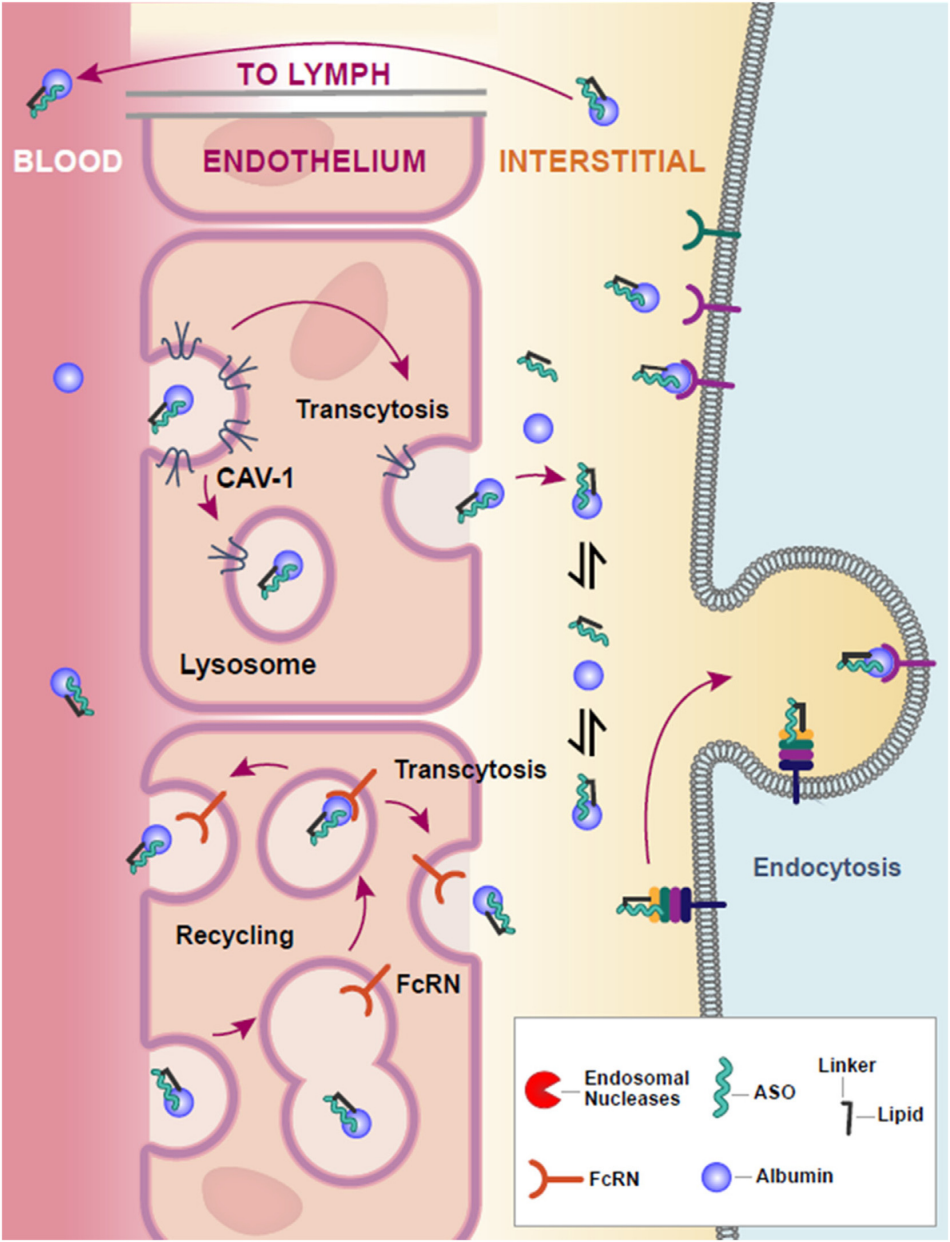
Model for the transport of Palm-ASO from circulation to target cells. Palm-ASO bound to albumin is transported across continuous endothelium through Cav-1 and FcRn-dependent transcytosis. Once in the interstitium, Palm-ASO can be endocytosed by target cells along with albumin; dissociate from albumin and be endocytosed independently; or transported through lymph, back to circulation.

Figure 7 depicts a model for the transport of Palm-ASO from circulation to target cells. Increasing affinity of ASO for albumin through conjugation with palmitic acid is effective in overcoming an early barrier to achieving knockdown in target cells and rapidly distributes ASO across the vascular endothelium to the interstitium of heart and skeletal muscle. However, this increased affinity prevents release of ASO in the interstitium and Palm-ASO remains sequestered in the interstitial fluid and is cleared from tissue with albumin through lymphatics. While conjugation with palmitate moderately improves ASO activity in muscle tissues, better improvements in activity might be achieved by further work investigating mechanisms to facilitate release of ASO in the interstitium. We observed quite robust accumulation of Palm-ASO in the interstitium of muscle tissues within 1–2 h of administration. If these conjugates can be improved to facilitate more robust uptake of these high concentrations of ASO by myocytes from the interstitium before the ASO is returned to circulation, we could potentially realize much better target knockdown.

## Supplementary Material

gkaa164_Supplemental_File
