# Supplementary material for: Mechanisms of palmitic acid-conjugated antisense oligonucleotide distribution in mice
Source: Nucleic Acids Res. 2020 Mar 17;48(8):4382–95. doi: 10.1093/nar/gkaa164 (PMC7192618; doi:10.1093/nar/gkaa164)
Supplement: gkaa164_Supplemental_File [file gkaa164_supplemental_file.pdf]

Supplemental Figure S1: Graphs of relative abundance of plasma proteins identified in mouse plasmas of different strains of mice

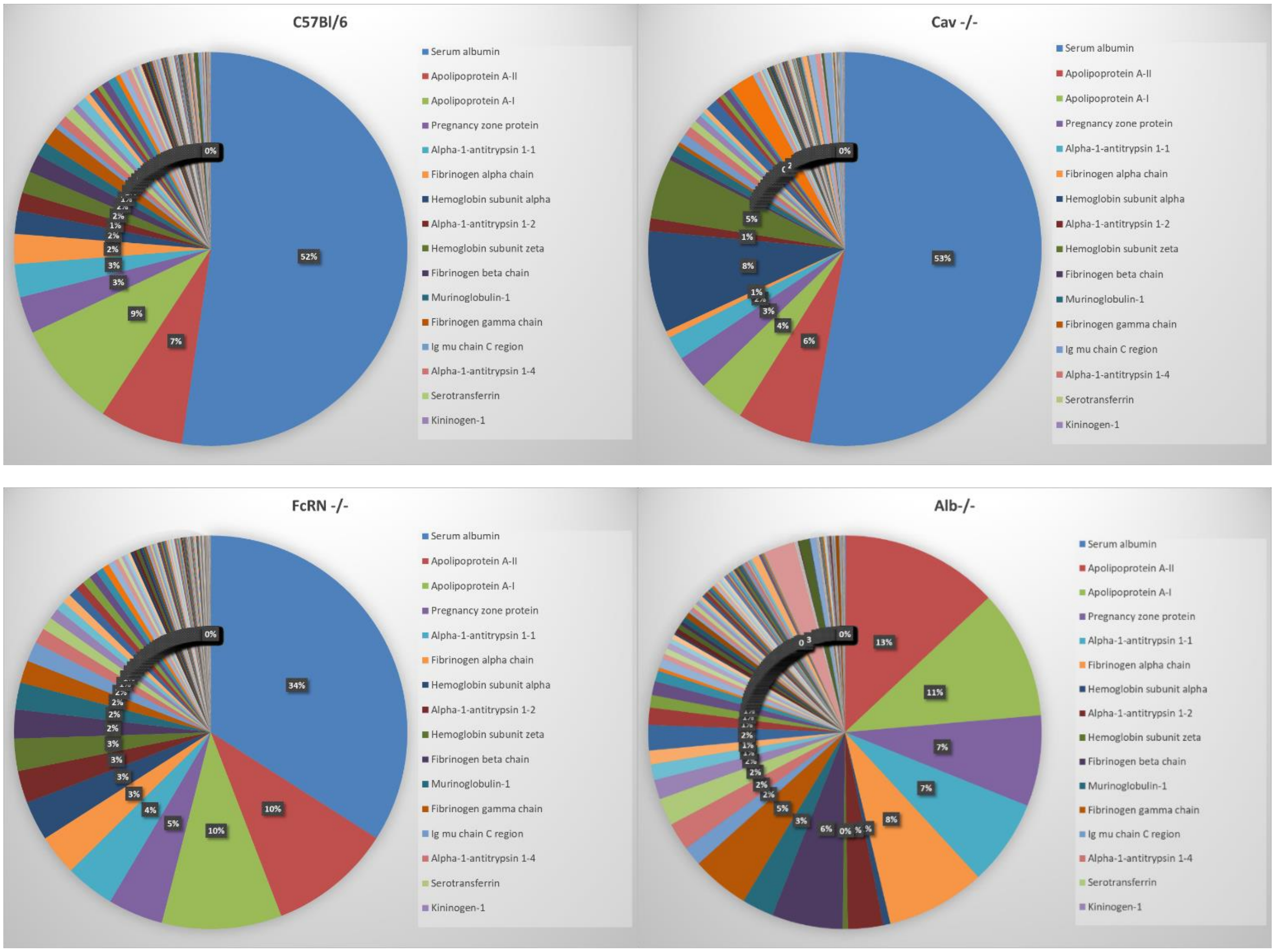

Supplemental Table S1: List of relative protein abundance

| Accession | Gene Name | Protein Name | C57Bl/6 | Cav-/- | FcRN-/- | Alb-/- |
|---|---|---|---|---|---|---|
| PO7724 | Alb | Serum albumin | 52.35 | 52.86 | 34.05 | 0.07 |
| P06728 | Apoa4 | Apolipoprotein A-II | 6.89 | 6.10 | 10.11 | 12.95 |
| P09813 | Apoa2 | Apolipoprotein A-I | 8.78 | 3.81 | 9.85 | 10.60 |
| P55302 | Lrpap1 | Pregnancy zone protein | 3.02 | 2.85 | 4.53 | 7.48 |
| P07758 | Serpina1a | Alpha-1-antitrypsin 1-1 | 2.72 | 1.95 | 4.05 | 7.05 |
| Q8K0E8 | Fgb | Fibrinogen alpha chain | 2.47 | 0.57 | 3.35 | 8.14 |
| P01942 | Hba | Hemoglobin subunit alpha | 1.91 | 8.33 | 3.25 | 0.58 |
| P22599 | Serpina1b | Alpha-1-antitrypsin 1-2 | 1.49 | 1.05 | 2.65 | 2.89 |
| P06467 | Hbz | Hemoglobin subunit zeta | 1.77 | 4.98 | 2.65 | 0.42 |
| Q8VCM7 | Fgg | Fibrinogen beta chain | 1.53 | 0.40 | 2.39 | 5.83 |
| P28665 | Mug1 | Murinoglobulin-1 | 1.17 | 0.93 | 2.21 | 2.61 |
| Q71KU9 | Fgl1 | Fibrinogen gamma chain | 1.44 | 0.32 | 1.84 | 4.75 |
| P01872 | Ighm | Ig mu chain C region | 0.44 | 0.77 | 1.61 | 1.58 |
| Q00897 | Serpina1d | Alpha-1-antitrypsin 1-4 | 0.78 | 0.74 | 1.28 | 2.28 |
| P05366 | Saa1 | Serotransferrin | 0.88 | 0.62 | 1.05 | 2.25 |
| O08677 | Kng1 | Kininogen-1 | 0.49 | 0.58 | 0.90 | 1.61 |
| Q00898 | Serpina1e | Alpha-1-antitrypsin 1-5 | 0.82 | 0.39 | 0.76 | 1.29 |
| P01887 | B2m | Beta-2-glycoprotein 1 | 0.48 | 0.39 | 0.76 | 1.14 |
| P01837 | N/A | Ig kappa chain C region | 0.37 | 1.02 | 0.74 | 2.09 |
| P33587 | Proc | Vitamin D-binding protein | 0.44 | 0.42 | 0.72 | 1.41 |
| E9Q414 | Apob | Apolipoprotein A-IV | 0.39 | 0.39 | 0.71 | 1.03 |
| P41317 | Mbl2 | Mannose-binding protein C | 0.62 | 0.53 | 0.68 | 1.13 |
| Q60590 | Orm1 | Alpha-1-acid glycoprotein 1 | 0.67 | 0.25 | 0.61 | 0.89 |
| P02088 | Hbb-b1 | Hemoglobin subunit beta-1 | 0.40 | 2.02 | 0.55 | 0.28 |
| Q9Z1R3 | Apom | Apolipoprotein E | 0.53 | 0.32 | 0.53 | 0.76 |
| Q91X72 | Hpx | Hemopexin | 0.46 | 0.34 | 0.38 | 0.43 |
| Q61233 | Lcp1 | Plasminogen | 0.21 | 0.16 | 0.29 | 0.48 |
| Q9CQI6 | Cotl1 | Clusterin | 0.31 | 0.12 | 0.29 | 0.48 |
| P03995 | Gfap | Gelsolin | 0.20 | 0.25 | 0.26 | 0.37 |
| P06909 | Cfh | Complement component C9 | 0.21 | 0.08 | 0.26 | 0.41 |
| P04945 | N/A | Ig kappa chain V-VI region NQ2-6.1 | 0.07 | 0.07 | 0.26 | 0.15 |
| Q00623 | Apoa1 | Antithrombin-III | 0.21 | 0.05 | 0.26 | 0.41 |
| P01029 | C4b | Complement C3 | 0.15 | 0.13 | 0.24 | 0.38 |
| Q01102 | Selp | Prothrombin | 0.18 | 0.10 | 0.22 | 0.31 |
| Q8VCT4 | Ces1d | Carboxylesterase 1C | 0.15 | 0.17 | 0.21 | 0.41 |
| Q921I1 | Tf | Serine protease inhibitor A3N | 0.16 | 0.09 | 0.19 | 0.47 |
| P01898 | H2-Q10 | H-2 class I histocompatibility antigen, Q10 alpha chain | 0.11 | 0.10 | 0.17 | 0.21 |
| E9PV24 | Fga | Fetuin-B | 0.12 | 0.13 | 0.16 | 0.34 |
| P14847 | Crp | Corticosteroid-binding globulin | 0.09 | 0.14 | 0.15 | 0.34 |
| P29788 | Vtn | Vitronectin | 0.18 | 0.14 | 0.15 | 0.30 |
| Q9WVJ3 | Cpq | Carboxypeptidase N subunit 2 | 0.12 | 0.09 | 0.15 | 0.20 |

| | | | | | | |
|---|---|---|---|---|---|---|
| O70165 | Fcn1 | Fibronectin | 0.10 | 0.08 | 0.14 | 0.30 |
| Q9ESB3 | Hrg | Histidine-rich glycoprotein | 0.08 | 0.08 | 0.14 | 0.23 |
| P06684 | C5 | Complement C4-B | 0.11 | 0.10 | 0.13 | 0.29 |
| P70274 | Sepp1 | Retinol-binding protein 4 | 0.10 | 0.06 | 0.13 | 0.14 |
| Q61176 | Arg1 | Apolipoprotein M | 0.11 | 0.10 | 0.13 | 0.16 |
| P47791 | Gsr | Glutathione peroxidase 3 | 0.09 | 0.09 | 0.12 | 0.23 |
| Q8BH61 | F13a1 | Coagulation factor XII | 0.08 | 0.08 | 0.12 | 0.22 |
| P06683 | C9 | Complement component C8 gamma chain | 0.09 | 0.05 | 0.12 | 0.18 |
| Q9D7Q1 | Chit1 | Ceruloplasmin | 0.07 | 0.08 | 0.11 | 0.20 |
| Q64726 | Azgp1 | Zinc-alpha-2-glycoprotein | 0.08 | 0.07 | 0.11 | 0.17 |
| P51910 | Apod | Apolipoprotein C-IV | 0.06 | 0.04 | 0.10 | 0.12 |
| P20918 | Plg | Plasma protease C1 inhibitor | 0.10 | 0.05 | 0.10 | 0.22 |
| Q8BH35 | C8b | Complement component C8 alpha chain | 0.10 | 0.05 | 0.10 | 0.20 |
| Q8VCG4 | C8g | Complement component C8 beta chain | 0.09 | 0.06 | 0.10 | 0.23 |
| Q61247 | Serpinf2 | Alpha-2-antiplasmin | 0.07 | 0.04 | 0.09 | 0.13 |
| P01592 | Jchain | Immunoglobulin J chain | 0.03 | 0.07 | 0.09 | 0.11 |
| Q9DBD0 | Ica | Inhibitor of carbonic anhydrase | 0.06 | 0.06 | 0.09 | 0.13 |
| Q61702 | Itih1 | Inter-alpha-trypsin inhibitor heavy chain H3 | 0.07 | 0.07 | 0.09 | 0.23 |
| O89020 | Afm | Afamin | 0.04 | 0.06 | 0.09 | 0.15 |
| P11680 | Cfp | Properdin | 0.06 | 0.05 | 0.08 | 0.14 |
| O70362 | Gpld1 | Phosphatidylinositol-glycan-specific phospholipase D | 0.06 | 0.05 | 0.08 | 0.10 |
| Q61704 | Itih3 | Inter-alpha-trypsin inhibitor heavy chain H2 | 0.05 | 0.07 | 0.08 | 0.21 |
| Q61730 | Il1rap | Interleukin-1 receptor accessory protein | 0.05 | 0.04 | 0.07 | 0.11 |
| P03987 | N/A | Ig gamma-3 chain C region | 0.09 | 0.14 | 0.07 | 0.20 |
| P39039 | Mbl1 | Mannose-binding protein A | 0.07 | 0.04 | 0.06 | 0.08 |
| A6X935 | Itih4 | Inter alpha-trypsin inhibitor, heavy chain 4 | 0.06 | 0.06 | 0.06 | 0.21 |
| P05367 | Saa2 | Serum amyloid A-2 protein | 0.06 | 0.00 | 0.06 | 0.24 |
| P49182 | Serpind1 | Heparin cofactor 2 | 0.04 | 0.03 | 0.06 | 0.08 |
| Q02105 | C1qc | Complement C1q subcomponent subunit B | 0.08 | 0.04 | 0.05 | 0.10 |
| P31532 | Saa4 | Serum amyloid A-1 protein | 0.05 | 0.00 | 0.05 | 0.23 |
| P01867 | Igh-3 | Ig gamma-2B chain C region | 0.14 | 0.39 | 0.05 | 0.66 |
| Q61268 | Apoc4 | Apolipoprotein B-100 | 0.05 | 0.03 | 0.05 | 0.12 |
| Q61703 | Itih2 | Inter-alpha-trypsin inhibitor heavy chain H1 | 0.03 | 0.03 | 0.05 | 0.10 |
| Q80YC5 | F12 | Coagulation factor X | 0.04 | 0.03 | 0.04 | 0.04 |
| P29621 | Serpina3c | Selenoprotein P | 0.03 | 0.02 | 0.04 | 0.05 |

| | | | | | | |
|---|---|---|---|---|---|---|
| Q9DBB9 | Cpn2 | Carboxypeptidase N catalytic chain | 0.03 | 0.03 | 0.04 | 0.07 |
| Q3UZ09 | C1rl | Complement C1q subcomponent subunit C | 0.03 | 0.03 | 0.04 | 0.11 |
| P58771 | Tpm1 | Transthyretin | 0.02 | 0.50 | 0.04 | 0.07 |
| P01864 | N/A | Ig gamma-2A chain C region secreted form | 0.11 | 0.43 | 0.04 | 2.51 |
| O08997 | Atox1 | Complement factor I | 0.03 | 0.03 | 0.04 | 0.07 |
| P51885 | Lum | Lumican | 0.03 | 0.03 | 0.04 | 0.06 |
| Q923D2 | Blvrb | Ficolin-1 | 0.02 | 0.02 | 0.03 | 0.06 |
| Q8VCS0 | Pglyrp2 | N-acetylmuramoyl-L-alanine amidase | 0.03 | 0.02 | 0.03 | 0.06 |
| P42703 | Lifr | Leukemia inhibitory factor receptor | 0.03 | 0.02 | 0.03 | 0.05 |
| P97290 | Serping1 | Plasma kallikrein | 0.02 | 0.02 | 0.03 | 0.06 |
| P12246 | Apcs | Serum amyloid A-4 protein | 0.28 | 0.14 | 0.03 | 0.80 |
| P16301 | Lcat | Phosphatidylcholine-sterol acyltransferase | 0.03 | 0.02 | 0.03 | 0.04 |
| P62737 | Acta2 | Actin, cytoplasmic 2 | 0.02 | 0.01 | 0.03 | 0.05 |
| P01635 | N/A | Ig kappa chain V-V region K2 (Fragment) | 0.01 | 0.01 | 0.03 | 0.02 |
| P01869 | Ighg1 | Ig gamma-1 chain C region, membrane-bound form | 0.25 | 0.60 | 0.03 | 0.52 |
| P21180 | C2 | Complement C1s-A subcomponent | 0.02 | 0.01 | 0.03 | 0.05 |
| Q9R098 | Hgfac | Hepatocyte growth factor activator | 0.02 | 0.02 | 0.03 | 0.05 |
| Q07968 | F13b | Coagulation factor XIII A chain | 0.02 | 0.01 | 0.03 | 0.05 |
| Q62087 | Pon3 | Serum paraoxonase/arylesterase 1 | 0.01 | 0.03 | 0.03 | 0.05 |
| P01750 | N/A | Ig heavy chain V region 102 | 0.02 | 0.04 | 0.03 | 0.05 |
| Q9JJN5 | Cpn1 | Carboxypeptidase B2 | 0.02 | 0.01 | 0.02 | 0.06 |
| Q8R121 | Serpina10 | Protein Z-dependent protease inhibitor | 0.01 | 0.01 | 0.02 | 0.04 |
| Q9Z0M6 | Cd97 | CD5 antigen-like | 0.01 | 0.02 | 0.02 | 0.03 |
| P16015 | Ca3 | Carbonic anhydrase 2 | 0.02 | 0.04 | 0.02 | 0.03 |
| P01636 | N/A | Ig kappa chain V-V region MOPC 149 | 0.01 | 0.02 | 0.02 | 0.09 |
| P01633 | Igk-V19-17 | Ig kappa chain V19-17 | 0.02 | 0.06 | 0.02 | 0.14 |
| P21460 | Cst3 | C-reactive protein | 0.02 | 0.01 | 0.02 | 0.04 |
| P21550 | Eno3 | Beta-2-microglobulin | 0.02 | 0.01 | 0.02 | 0.02 |
| P14106 | C1qb | Complement C1q subcomponent subunit A | 0.01 | 0.01 | 0.02 | 0.04 |
| P47878 | Igfbp3 | Insulin-like growth factor-binding protein 3 | 0.00 | 0.00 | 0.02 | 0.01 |
| P01786 | N/A | Ig heavy chain V region MOPC 47A | 0.02 | 0.05 | 0.02 | 0.08 |

| | | | | | | |
|---|---|---|---|---|---|---|
| P18760 | Cfl1 | Coagulation factor XIII B chain | 0.01 | 0.01 | 0.02 | 0.03 |
| P97298 | Serpinf1 | Phospholipid transfer protein | 0.02 | 0.02 | 0.02 | 0.04 |
| Q9DCW4 | Etfb | EGF-containing fibulin-like extracellular matrix protein 1 | 0.01 | 0.01 | 0.02 | 0.03 |
| P47880 | Igfbp6 | Insulin-like growth factor-binding protein complex acid labile subunit | 0.01 | 0.01 | 0.02 | 0.02 |
| P01631 | N/A | Ig kappa chain V-II region 26-10 | 0.01 | 0.02 | 0.02 | 0.05 |
| P35505 | Fah | Fructose-bisphosphate aldolase A | 0.00 | 0.01 | 0.01 | 0.03 |
| P04202 | Tgfb1 | Transcobalamin-2 | 0.01 | 0.01 | 0.01 | 0.02 |
| Q07456 | Ambp | Protein AMBP | 0.01 | 0.01 | 0.01 | 0.03 |
| Q91WP6 | Serpina3n | Serine protease inhibitor A3M | 0.05 | 0.01 | 0.01 | 0.11 |
| Q8K182 | C8a | Complement C5 | 0.01 | 0.01 | 0.01 | 0.02 |
| O09164 | Sod3 | Extracellular matrix protein 1 | 0.01 | 0.01 | 0.01 | 0.02 |
| P01643 | N/A | Ig kappa chain V-V region MOPC 173 | 0.01 | 0.01 | 0.01 | 0.01 |
| O08742 | Gp5 | Platelet glycoprotein Ib alpha chain | 0.00 | 0.01 | 0.01 | 0.02 |
| Q9JM99 | Prg4 | Vitamin K-dependent protein C | 0.01 | 0.01 | 0.01 | 0.02 |
| P09581 | Csf1r | Macrophage colony-stimulating factor 1 receptor | 0.01 | 0.01 | 0.01 | 0.02 |
| P17182 | Eno1 | Alpha-amylase 1 | 0.01 | 0.01 | 0.01 | 0.02 |
| P26262 | Klkb1 | Pigment epithelium-derived factor | 0.01 | 0.01 | 0.01 | 0.02 |
| Q91Y97 | Aldob | Fructose-bisphosphate aldolase B | 0.00 | 0.00 | 0.01 | 0.01 |
| Q9DC11 | Plxdc2 | Platelet-activating factor acetylhydrolase | 0.01 | 0.00 | 0.01 | 0.02 |
| P07361 | Orm2 | Alpha-1-acid glycoprotein 2 | 0.01 | 0.01 | 0.01 | 0.04 |
| P60843 | Eif4a1 | Epidermal growth factor receptor | 0.01 | 0.01 | 0.01 | 0.00 |
| Q9DCT8 | Crip2 | Cystatin-C | 0.01 | 0.01 | 0.01 | 0.01 |
| P32261 | Serpinc1 | Angiotensinogen | 0.01 | 0.01 | 0.01 | 0.02 |
| P70375 | F7 | Coagulation factor V | 0.01 | 0.01 | 0.01 | 0.02 |
| Q60994 | Adipoq | Adiponectin | 0.00 | 0.01 | 0.01 | 0.01 |
| P01680 | N/A | Ig kappa chain V-IV region S107B | 0.01 | 0.01 | 0.01 | 0.03 |
| P05017 | Igf1 | Insulin-like growth factor I | 0.00 | 0.00 | 0.01 | 0.01 |
| P08226 | Apoe | Apolipoprotein D | 0.01 | 0.01 | 0.01 | 0.00 |
| P18337 | Sell | L-selectin | 0.00 | 0.00 | 0.01 | 0.01 |
| Q93092 | Taldo1 | Thyroxine-binding globulin | 0.00 | 0.00 | 0.01 | 0.02 |
| P97464 | Ext1 | Eukaryotic translation initiation factor 3 subunit E | 0.01 | 0.01 | 0.01 | 0.00 |
| Q9R0G6 | Comp | Carboxypeptidase Q | 0.00 | 0.01 | 0.01 | 0.01 |
| O88783 | F5 | Coagulation factor IX | 0.00 | 0.00 | 0.01 | 0.01 |
| P26040 | Ezr | Extracellular superoxide dismutase [Cu-Zn] | 0.00 | 0.01 | 0.01 | 0.01 |
| P00920 | Ca2 | Carbonic anhydrase 1 | 0.00 | 0.01 | 0.01 | 0.00 |
| Q61171 | Prdx2 | Peroxiredoxin-2 | 0.00 | 0.01 | 0.01 | 0.01 |

| | | | | | | |
|---|---|---|---|---|---|---|
| Q61646 | Hp | Haptoglobin | 0.02 | 0.00 | 0.01 | 0.29 |
| P01630 | N/A | Ig kappa chain V-II region 7S34.1 | 0.01 | 0.20 | 0.01 | 0.04 |
| P26339 | Chga | Cholinesterase | 0.00 | 0.00 | 0.01 | 0.01 |
| P07214 | Sparc | Sorbitol dehydrogenase | 0.00 | 0.00 | 0.01 | 0.01 |
| Q08761 | Pros1 | Vitamin k-dependent protein s | 0.00 | 0.00 | 0.01 | 0.01 |
| P08228 | Sod1 | Sulfhydryl oxidase 1 | 0.00 | 0.00 | 0.01 | 0.01 |
| P98064 | Masp1 | Mannan-binding lectin serine protease 1 | 0.01 | 0.00 | 0.00 | 0.01 |
| O88947 | F10 | Coagulation factor VII | 0.01 | 0.00 | 0.00 | 0.01 |
| P35455 | Avp | Vascular cell adhesion protein 1 | 0.00 | 0.00 | 0.00 | 0.01 |
| P26043 | Rdx | Pyruvate kinase PKM | 0.00 | 0.00 | 0.00 | 0.01 |
| Q61129 | Cfi | Complement factor H | 0.00 | 0.00 | 0.00 | 0.01 |
| P19221 | F2 | Proteoglycan 4 | 0.00 | 0.00 | 0.00 | 0.02 |
| P55284 | Cdh5 | Cadherin-1 | 0.00 | 0.00 | 0.00 | 0.00 |
| P99026 | Psmb4 | Proteasome subunit beta type-4 | 0.00 | 0.00 | 0.00 | 0.01 |
| Q06318 | Scgb1a1 | Uromodulin | 0.00 | 0.00 | 0.00 | 0.00 |
| Q9R1P4 | Psma1 | Proteasome subunit alpha type-6 | 0.00 | 0.00 | 0.00 | 0.01 |
| P16045 | Lgals1 | Fumarylacetoacetase | 0.00 | 0.00 | 0.00 | 0.00 |
| Q9CY50 | Ssr1 | Transketolase | 0.00 | 0.00 | 0.00 | 0.01 |
| Q01149 | Col1a2 | Collagen alpha-1(XII) chain | 0.00 | 0.00 | 0.00 | 0.00 |
| P52480 | Pkm | Pyruvate kinase PKLR | 0.00 | 0.00 | 0.00 | 0.01 |
| P09803 | Cdh1 | BPI fold-containing family A member 2 | 0.00 | 0.00 | 0.00 | 0.03 |
| Q9Z126 | Pf4 | Plastin-2 | 0.00 | 0.00 | 0.00 | 0.01 |
| Q7TQ62 | Podn | Plexin domain-containing protein 2 | 0.00 | 0.00 | 0.00 | 0.00 |
| P07743 | Bpifa2 | Bleomycin hydrolase | 0.00 | 0.00 | 0.00 | 0.01 |
| P98086 | C1qa | Collectin-11 | 0.00 | 0.00 | 0.00 | 0.00 |
| Q8C255 | Dpep2 | Di-N-acetylchitobiase | 0.00 | 0.00 | 0.00 | 0.00 |
| Q62356 | Fstl1 | Flavin reductase (NADPH) | 0.00 | 0.01 | 0.00 | 0.00 |
| Q9CPY7 | Lap3 | Cytochrome c, somatic | 0.00 | 0.00 | 0.00 | 0.01 |
| P63260 | Actg1 | Actin, aortic smooth muscle | 0.00 | 0.00 | 0.00 | 0.01 |
| P03976 | N/A | Ig kappa chain V-II region 17S29.1 | 0.00 | 0.00 | 0.00 | 0.03 |
| P13597 | Icam1 | Intercellular adhesion molecule 1 | 0.00 | 0.00 | 0.00 | 0.00 |
| P18242 | Ctsd | Cathepsin B | 0.00 | 0.00 | 0.00 | 0.00 |
| Q9WUU7 | Ctsz | Cathepsin S | 0.00 | 0.00 | 0.00 | 0.01 |
| Q03734 | Serpina3m | Serine protease inhibitor A3C | 0.00 | 0.00 | 0.00 | 0.00 |
| Q99K82 | Smox | SPARC-like protein 1 | 0.00 | 0.00 | 0.00 | 0.00 |
| Q61838 | Pzp | Podocan | 0.00 | 0.00 | 0.00 | 0.00 |
| Q9Z2U1 | Psma5 | Proteasome subunit alpha type-2 | 0.00 | 0.00 | 0.00 | 0.00 |
| P08121 | Col3a1 | Collagen alpha-1(I) chain | 0.00 | 0.00 | 0.00 | 0.00 |
| O88998 | Olfm1 | Noelin | 0.00 | 0.00 | 0.00 | 0.00 |
| Q8K135 | Kiaa0319l | Dipeptidase 2 | 0.00 | 0.00 | 0.00 | 0.00 |
| P13595 | Ncam1 | Neural cell adhesion molecule 1 | 0.00 | 0.00 | 0.00 | 0.00 |
| Q91V98 | Cd248 | Endoplasmic reticulum aminopeptidase 1 | 0.00 | 0.00 | 0.00 | 0.00 |

| | | | | | | |
|---|---|---|---|---|---|---|
| Q9Z1T2 | Thbs4 | Thrombospondin-1 | 0.00 | 0.00 | 0.00 | 0.00 |
| P29533 | Vcam1 | Uteroglobin | 0.00 | 0.00 | 0.00 | 0.00 |
| Q7TPR4 | Actn1 | Alpha-2-macroglobulin-P | 0.00 | 0.00 | 0.00 | 0.00 |
| P08071 | Ltf | Lactotransferrin | 0.00 | 0.00 | 0.00 | 0.00 |
| Q9QWK4 | Cd5l | Cathepsin Z | 0.00 | 0.00 | 0.00 | 0.00 |
| Q9CQF9 | Pcyox1 | Prenylcysteine oxidase | 0.00 | 0.00 | 0.00 | 0.00 |
| P09528 | Fth1 | Ezrin | 0.00 | 0.00 | 0.00 | 0.00 |
| Q8R016 | Blmh | Bisphosphoglycerate mutase | 0.00 | 0.00 | 0.00 | 0.00 |
| Q06890 | Clu | Chromogranin-A | 0.00 | 0.00 | 0.00 | 0.00 |
| Q8QZR4 | Oaf | Out at first protein homolog | 0.00 | 0.00 | 0.00 | 0.00 |
| P58802 | Tbc1d10a | Superoxide dismutase [Cu-Zn] | 0.00 | 0.00 | 0.00 | 0.00 |
| P62897 | Cycs | Cysteine-rich protein 2 | 0.00 | 0.00 | 0.00 | 0.00 |
| P00688 | Amy2 | Pancreatic alpha-amylase | 0.00 | 0.00 | 0.00 | 0.00 |
| Q9JHH6 | Cpb2 | Carboxylesterase 3A | 0.00 | 0.00 | 0.00 | 0.00 |
| Q8K0D2 | Habp2 | Hyaluronan-binding protein 2 | 0.00 | 0.00 | 0.00 | 0.00 |
| P10923 | Spp1 | Osteopontin | 0.00 | 0.00 | 0.00 | 0.00 |
| P35700 | Prdx1 | Peroxiredoxin-1 | 0.00 | 0.00 | 0.00 | 0.00 |
| Q9DBP5 | Cmpk1 | Ubiquitin-like protein ISG15 | 0.00 | 0.00 | 0.00 | 0.00 |
| P01674 | N/A | Ig kappa chain V-III region PC 2154 | 0.00 | 0.01 | 0.00 | 0.01 |
| Q02819 | Nucb1 | Nucleobindin-1 | 0.00 | 0.00 | 0.00 | 0.00 |
| Q01339 | Apoh | Beta-1,4-galactosyltransferase 1 | 0.00 | 0.00 | 0.00 | 0.00 |
| Q9QUM9 | Psma6 | Proteasome subunit alpha type-1 | 0.00 | 0.00 | 0.00 | 0.00 |
| Q91WP0 | Masp2 | Mannan-binding lectin serine protease 2 | 0.00 | 0.00 | 0.00 | 0.00 |
| P10404 | N/A | MLV-related proviral Env polyprotein | 0.00 | 0.00 | 0.00 | 0.00 |
| P49722 | Psma2 | Proteasome subunit alpha type-4 | 0.00 | 0.00 | 0.00 | 0.00 |
| O88968 | Tcn2 | Transaldolase | 0.00 | 0.00 | 0.00 | 0.00 |
| Q9R097 | Spint1 | Kunitz-type protease inhibitor 1 | 0.00 | 0.00 | 0.00 | 0.00 |
| P56480 | Atp5b | Arginase-1 | 0.00 | 0.00 | 0.00 | 0.00 |
| Q9Z0M9 | Il18bp | Interleukin-18-binding protein | 0.00 | 0.00 | 0.00 | 0.00 |
| Q9R1P0 | Psma4 | Proteasome subunit alpha type-5 | 0.00 | 0.00 | 0.00 | 0.00 |
| P10493 | Nid1 | Nidogen-1 | 0.00 | 0.00 | 0.00 | 0.00 |
| P12399 | Ctla2a | Protein CTLA-2-alpha | 0.00 | 0.00 | 0.00 | 0.00 |
| Q91ZX7 | Lrp1 | Prolow-density lipoprotein receptor-related protein 1 | 0.00 | 0.00 | 0.00 | 0.00 |
| P55065 | Pltp | Glycerol-3-phosphate phosphatase | 0.00 | 0.00 | 0.00 | 0.00 |
| P21614 | Gc | Vinculin | 0.00 | 0.00 | 0.00 | 0.00 |
| Q9D1H9 | Mfap4 | Microfibril-associated glycoprotein 4 | 0.00 | 0.00 | 0.00 | 0.00 |
| O35930 | Gp1ba | Platelet factor 4 | 0.00 | 0.00 | 0.00 | 0.00 |
| O08709 | Prdx6 | Peroxiredoxin-6 | 0.00 | 0.00 | 0.00 | 0.00 |
| Q60963 | Pla2g7 | Platelet glycoprotein V | 0.00 | 0.00 | 0.00 | 0.00 |
| Q64727 | Vcl | Vasorin | 0.00 | 0.00 | 0.00 | 0.00 |

| | | | | | | |
|---|---|---|---|---|---|---|
| P11438 | Lamp1 | Lysosome-associated membrane glycoprotein 1 | 0.00 | 0.00 | 0.00 | 0.00 |
| P13020 | Gsn | Gamma-glutamyl hydrolase | 0.00 | 0.00 | 0.00 | 0.00 |
| Q61468 | Msln | Mesothelin | 0.00 | 0.00 | 0.00 | 0.00 |
| P70389 | Igfals | Insulin-like growth factor-binding protein 6 | 0.00 | 0.00 | 0.00 | 0.00 |
| Q9CWJ9 | Atic | Beta-enolase | 0.00 | 0.00 | 0.00 | 0.00 |
| O08689 | Mstn | Growth/differentiation factor 8 | 0.00 | 0.00 | 0.00 | 0.00 |
| Q00724 | Rbp4 | Retinoic acid receptor responder protein 2 | 0.00 | 0.00 | 0.00 | 0.00 |
| Q9CY02 | Ahsp | Alpha-enolase | 0.00 | 0.00 | 0.00 | 0.00 |
| Q8R1U2 | Cgref1 | CD97 antigen | 0.00 | 0.00 | 0.00 | 0.00 |
| P53657 | Pklr | P-selectin | 0.00 | 0.00 | 0.00 | 0.00 |
| P70296 | Pebp1 | Phosphatidylethanolamine-binding protein 1 | 0.00 | 0.00 | 0.00 | 0.00 |
| P62962 | Pfn1 | Profilin-1 | 0.00 | 0.00 | 0.00 | 0.00 |
| P10639 | Txn | Tenascin | 0.00 | 0.00 | 0.00 | 0.00 |
| Q9Z0L8 | Ggh | Galectin-1 | 0.00 | 0.00 | 0.00 | 0.00 |
| O09159 | Man2b1 | Lysosomal alpha-mannosidase | 0.00 | 0.00 | 0.00 | 0.00 |
| P35330 | Icam2 | Intercellular adhesion molecule 2 | 0.00 | 0.00 | 0.00 | 0.00 |
| P16125 | Ldhb | L-lactate dehydrogenase b chain | 0.00 | 0.00 | 0.00 | 0.00 |
| P28798 | Grn | Granulins | 0.00 | 0.00 | 0.00 | 0.00 |
| O55042 | Snca | Alpha-hemoglobin-stabilizing protein | 0.00 | 0.00 | 0.00 | 0.00 |
| P31725 | S100a9 | Protein S100-A9 | 0.00 | 0.00 | 0.00 | 0.00 |
| P62827 | Ran | GTP-binding nuclear protein Ran | 0.00 | 0.00 | 0.00 | 0.00 |
| Q8CHP8 | Pgp | Glycerol-3-phosphate dehydrogenase [NAD(+)], cytoplasmic | 0.00 | 0.00 | 0.00 | 0.00 |
| Q9CPU0 | Glo1 | Lactoylglutathione lyase | 0.00 | 0.00 | 0.00 | 0.00 |
| O35955 | Psmb10 | Proteasome subunit beta type-10 | 0.00 | 0.00 | 0.00 | 0.00 |
| O70370 | Ctss | Cathepsin D | 0.00 | 0.00 | 0.00 | 0.00 |
| Q64442 | Sord | Serum paraoxonase/lactonase 3 | 0.00 | 0.00 | 0.00 | 0.00 |
| P10605 | Ctsb | Cartilage oligomeric matrix protein | 0.00 | 0.00 | 0.00 | 0.00 |
| Q80W65 | Pcsk9 | Proprotein convertase subtilisin/kexin type 9 | 0.00 | 0.00 | 0.00 | 0.00 |
| Q01279 | Egfr | Endosialin | 0.00 | 0.00 | 0.00 | 0.00 |
| P05064 | Aldoa | Follistatin-related protein 1 | 0.00 | 0.00 | 0.00 | 0.00 |
| Q9ET01 | Pygl | Glycogen phosphorylase, liver form | 0.00 | 0.00 | 0.00 | 0.00 |
| O70251 | Eef1b | Elongation factor 1-alpha 1 | 0.00 | 0.00 | 0.00 | 0.00 |
| P21570 | Ang | Aminoacyl tRNA synthase complex-interacting multifunctional protein 1 | 0.00 | 0.00 | 0.00 | 0.00 |
| O09061 | Psmb1 | Proteasome subunit beta type-1 | 0.00 | 0.00 | 0.00 | 0.00 |

| | | | | | | |
|---|---|---|---|---|---|---|
| Q63918 | Sdpr | Serum amyloid P-component | 0.00 | 0.00 | 0.00 | 0.00 |
| Q61398 | Pcolce | Procollagen C-endopeptidase enhancer 1 | 0.00 | 0.00 | 0.00 | 0.00 |
| P51125 | Cast | Cadherin-5 | 0.00 | 0.00 | 0.00 | 0.00 |
| O70456 | Sfn | 14-3-3 protein sigma | 0.00 | 0.00 | 0.00 | 0.00 |
| P16675 | Ctsa | Lysosomal protective protein | 0.00 | 0.00 | 0.00 | 0.00 |
| Q91X17 | Umod | UMP-CMP kinase | 0.00 | 0.00 | 0.00 | 0.00 |
| Q6P253 | Dmkn | Cytosol aminopeptidase | 0.00 | 0.00 | 0.00 | 0.00 |
| Q64739 | Col11a2 | Collagen alpha-2(I) chain | 0.00 | 0.00 | 0.00 | 0.00 |
| P11499 | Hsp90ab1 | Heat shock protein HSP 90-beta | 0.00 | 0.00 | 0.00 | 0.00 |
| Q61147 | Cp | Cell growth regulator with EF hand domain protein 1 | 0.00 | 0.00 | 0.00 | 0.00 |
| Q02858 | Tek | Angiogenin | 0.00 | 0.00 | 0.00 | 0.00 |
| P70663 | Sparcl1 | SPARC | 0.00 | 0.00 | 0.00 | 0.00 |
| Q8R4K8 | Pappa | Pappalysin-1 | 0.00 | 0.00 | 0.00 | 0.00 |
| Q8CCK0 | H2afy2 | Copper transport protein ATOX1 | 0.00 | 0.00 | 0.00 | 0.00 |
| Q9D8Y0 | Efhd2 | Dystroglycan | 0.00 | 0.00 | 0.00 | 0.00 |
| Q9D358 | Acp1 | Low molecular weight phosphotyrosine protein phosphatase | 0.00 | 0.00 | 0.00 | 0.00 |
| Q9EPX2 | Papln | Papilin | 0.00 | 0.00 | 0.00 | 0.00 |
| Q9D819 | Ppa1 | Inorganic pyrophosphatase | 0.00 | 0.00 | 0.00 | 0.00 |
| Q9DBG5 | Plin3 | Perilipin-3 | 0.00 | 0.00 | 0.00 | 0.00 |
| P13707 | Gpd1 | Glutathione S-transferase Mu 1 | 0.00 | 0.00 | 0.00 | 0.00 |
| Q6GQT1 | A2m | Alpha-2-macroglobulin receptor-associated protein | 0.00 | 0.00 | 0.00 | 0.00 |
| P10649 | Gstm1 | Glutathione S-transferase A3 | 0.00 | 0.00 | 0.00 | 0.00 |
| Q3UM45 | Ppp1r7 | Protein phosphatase 1 regulatory subunit 7 | 0.00 | 0.00 | 0.00 | 0.00 |
| Q9CQ60 | Pgls | 6-phosphogluconolactonase | 0.00 | 0.00 | 0.00 | 0.00 |
| Q78PG9 | Ccdc25 | Cofilin-1 | 0.00 | 0.00 | 0.00 | 0.00 |
| P62806 | Hist1h4a | Histone H4 | 0.00 | 0.00 | 0.00 | 0.00 |
| Q8CG14 | C1sa | Complement C1r subcomponent-like protein | 0.00 | 0.00 | 0.00 | 0.00 |
| Q62165 | Dag1 | Dyslexia-associated protein KIAA0319-like protein | 0.00 | 0.00 | 0.00 | 0.00 |
| Q03311 | Bche | Chitotriosidase-1 | 0.00 | 0.00 | 0.00 | 0.00 |
| Q02596 | Glycam1 | Glycosylation-dependent cell adhesion molecule 1 | 0.00 | 0.00 | 0.00 | 0.00 |
| Q9QXC1 | Fetub | Ferritin heavy chain | 0.00 | 0.00 | 0.00 | 0.00 |
| P00687 | Amy1 | Alpha-actinin-1 | 0.00 | 0.00 | 0.00 | 0.00 |
| P11276 | Fn1 | Fibrinogen-like protein 1 | 0.00 | 0.00 | 0.00 | 0.00 |
| P63038 | Hspd1 | 60 kDa heat shock protein, mitochondrial | 0.00 | 0.00 | 0.00 | 0.00 |
| Q9CZT5 | Vasn | Vasopressin-neurophysin 2-copeptin | 0.00 | 0.00 | 0.00 | 0.00 |

| | | | | | | |
|---|---|---|---|---|---|---|
| P21107 | Tpm3 | Tropomyosin alpha-1 chain | 0.00 | 0.00 | 0.00 | 0.00 |
| P61939 | Serpina7 | Thrombospondin-4 | 0.00 | 0.00 | 0.00 | 0.00 |
| O88685 | Psmc3 | 26S protease regulatory subunit 6A | 0.00 | 0.00 | 0.00 | 0.00 |
| Q9CR00 | Psmd9 | 26S proteasome non-ATPase regulatory subunit 9 | 0.00 | 0.00 | 0.00 | 0.00 |
| P25444 | Rps2 | 40S ribosomal protein S2 | 0.00 | 0.00 | 0.00 | 0.00 |
| P60867 | Rps20 | 40S ribosomal protein S20 | 0.00 | 0.00 | 0.00 | 0.00 |
| P20029 | Hspa5 | 78 kDa glucose-regulated protein | 0.00 | 0.00 | 0.00 | 0.00 |
| Q80T21 | Adamtsl4 | ADAMTS-like protein 4 | 0.00 | 0.00 | 0.00 | 0.00 |
| Q8R2Z0 | Fam132a | Adipolin | 0.00 | 0.00 | 0.00 | 0.00 |
| Q19LI2 | A1bg | Alpha-1B-glycoprotein | 0.00 | 0.02 | 0.00 | 0.00 |
| P31230 | Aimp1 | Alpha-synuclein | 0.00 | 0.00 | 0.00 | 0.00 |
| P11859 | Agt | Angiopoietin-1 receptor | 0.00 | 0.00 | 0.00 | 0.00 |
| Q9DCX2 | Atp5h | ATP synthase subunit beta, mitochondrial | 0.00 | 0.00 | 0.00 | 0.00 |
| P18293 | Npr1 | ATP synthase subunit d, mitochondrial | 0.00 | 0.00 | 0.00 | 0.00 |
| P15535 | B4galt1 | Atrial natriuretic peptide receptor 1 | 0.00 | 0.00 | 0.00 | 0.00 |
| P15327 | Bpgm | Bifunctional purine biosynthesis protein PURH | 0.00 | 0.00 | 0.00 | 0.00 |
| Q3UZA1 | Rcsd1 | Calpastatin | 0.00 | 0.00 | 0.00 | 0.00 |
| P13634 | Ca1 | CapZ-interacting protein | 0.00 | 0.00 | 0.00 | 0.00 |
| P23953 | Ces1c | Carbonic anhydrase 3 | 0.00 | 0.00 | 0.00 | 0.00 |
| Q63880 | Ces3a | Carboxylesterase 1D | 0.00 | 0.00 | 0.00 | 0.00 |
| P16294 | F9 | Coactosin-like protein | 0.00 | 0.00 | 0.00 | 0.00 |
| P11087 | Col1a1 | Coiled-coil domain-containing protein 25 | 0.00 | 0.00 | 0.00 | 0.00 |
| Q60847 | Col12a1 | Collagen alpha-1(III) chain | 0.00 | 0.00 | 0.00 | 0.00 |
| Q3SXB8 | Colec11 | Collagen alpha-2(XI) chain | 0.00 | 0.00 | 0.00 | 0.00 |
| P01027 | C3 | Complement C2 | 0.00 | 0.00 | 0.00 | 0.00 |
| Q9WUM4 | Coro1c | Core histone macro-H2A.2 | 0.00 | 0.00 | 0.00 | 0.00 |
| Q06770 | Serpina6 | Coronin-1C | 0.00 | 0.00 | 0.00 | 0.00 |
| O55111 | Dsg2 | Dermokine | 0.00 | 0.00 | 0.00 | 0.00 |
| Q8R242 | Ctbs | Desmoglein-2 | 0.00 | 0.00 | 0.00 | 0.00 |
| Q8BPB5 | Efemp1 | EF-hand domain-containing protein D2 | 0.00 | 0.00 | 0.00 | 0.00 |
| P10126 | Eef1a1 | Electron transfer flavoprotein subunit beta | 0.00 | 0.00 | 0.00 | 0.00 |
| Q9EQH2 | Erap1 | Elongation factor 1-beta | 0.00 | 0.00 | 0.00 | 0.00 |
| P60229 | Eif3e | Eukaryotic initiation factor 4A-I | 0.00 | 0.00 | 0.00 | 0.00 |
| Q9QZD9 | Eif3i | Eukaryotic translation initiation factor 3 subunit I | 0.00 | 0.00 | 0.00 | 0.00 |
| Q8BGD9 | Eif4b | Eukaryotic translation initiation factor 4B | 0.00 | 0.00 | 0.00 | 0.00 |

| | | | | | | |
|---|---|---|---|---|---|---|
| Q61508 | Ecm1 | Exostosin-1 | 0.00 | 0.00 | 0.00 | 0.00 |
| O08795 | Prkcsh | Glial fibrillary acidic protein | 0.00 | 0.00 | 0.00 | 0.00 |
| P35436 | Grin2a | Glucosidase 2 subunit beta | 0.00 | 0.00 | 0.00 | 0.00 |
| P15105 | Glul | Glutamate receptor ionotropic, NMDA 2A | 0.00 | 0.00 | 0.00 | 0.00 |
| P46412 | Gpx3 | Glutamine synthetase | 0.00 | 0.00 | 0.00 | 0.00 |
| P30115 | Gsta3 | Glutathione reductase, mitochondrial | 0.00 | 0.00 | 0.00 | 0.00 |
| Q8BH60 | Gopc | Golgi-associated PDZ and coiled-coil motif-containing protein | 0.00 | 0.00 | 0.00 | 0.00 |
| Q9R257 | Hebp1 | Heme-binding protein 1 | 0.00 | 0.00 | 0.00 | 0.00 |
| P01878 | N/A | Ig alpha chain C region | 0.00 | 0.00 | 0.00 | 0.00 |
| P09535 | Igf2 | Insulin-like growth factor II | 0.00 | 0.00 | 0.00 | 0.00 |
| P06796 | Csn3 | Kappa-casein | 0.00 | 0.00 | 0.00 | 0.00 |
| Q61781 | Krt14 | Keratin, type I cytoskeletal 14 | 0.00 | 0.00 | 0.00 | 0.00 |
| Q9Z2K1 | Krt16 | Keratin, type I cytoskeletal 16 | 0.00 | 0.00 | 0.00 | 0.00 |
| Q922U2 | Krt5 | Keratin, type II cytoskeletal 2 epidermal | 0.00 | 0.00 | 0.00 | 0.00 |
| Q3TTY5 | Krt2 | Keratin, type II cytoskeletal 2 oral | 0.00 | 0.00 | 0.00 | 0.00 |
| P11679 | Krt8 | Keratin, type II cytoskeletal 5 | 0.00 | 0.00 | 0.00 | 0.00 |
| Q3UV17 | Krt76 | Keratin, type II cytoskeletal 8 | 0.00 | 0.00 | 0.00 | 0.00 |
| Q60675 | Lama2 | Laminin subunit alpha-2 | 0.00 | 0.00 | 0.00 | 0.00 |
| P48356 | Lepr | Leptin receptor | 0.00 | 0.00 | 0.00 | 0.00 |
| Q8BSS9 | Ppfia2 | Liprin-alpha-2 | 0.00 | 0.00 | 0.00 | 0.00 |
| P34884 | Mif | Macrophage migration inhibitory factor | 0.00 | 0.00 | 0.00 | 0.00 |
| Q9R0S3 | Mmp17 | Matrix metalloproteinase-17 | 0.00 | 0.00 | 0.00 | 0.00 |
| P25785 | Timp2 | Metalloproteinase inhibitor 2 | 0.00 | 0.00 | 0.00 | 0.00 |
| P11247 | Mpo | Myeloperoxidase | 0.00 | 0.00 | 0.00 | 0.00 |
| Q60605 | Myl6 | Myosin light polypeptide 6 | 0.00 | 0.00 | 0.00 | 0.00 |
| Q9CWS0 | Ddah1 | N(G),N(G)-dimethylarginine dimethylaminohydrolase 1 | 0.00 | 0.00 | 0.00 | 0.00 |
| Q8VDK1 | Nit1 | Nitrilase homolog 1 | 0.00 | 0.00 | 0.00 | 0.00 |
| Q78ZA7 | Nap1l4 | Nucleosome assembly protein 1-like 4 | 0.00 | 0.00 | 0.00 | 0.00 |
| Q9QUR7 | Pin1 | Peptidyl-prolyl cis-trans isomerase NIMA-interacting 1 | 0.00 | 0.00 | 0.00 | 0.00 |
| O08807 | Prdx4 | Peroxiredoxin-4 | 0.00 | 0.00 | 0.00 | 0.00 |
| Q80XD8 | Prap1 | Proline-rich acidic protein 1 | 0.00 | 0.00 | 0.00 | 0.00 |
| P97372 | Psme2 | Proteasome activator complex subunit 2 | 0.00 | 0.00 | 0.00 | 0.00 |
| Q7TNV0 | Dek | Protein DEK | 0.00 | 0.00 | 0.00 | 0.00 |
| P09103 | P4hb | Protein disulfide-isomerase | 0.00 | 0.00 | 0.00 | 0.00 |
| P50543 | S100a11 | Protein S100-A11 | 0.00 | 0.00 | 0.00 | 0.00 |
| Q9DD06 | Rarres2 | Radixin | 0.00 | 0.00 | 0.00 | 0.00 |

| | | | | | | |
|---|---|---|---|---|---|---|
| P52430 | Pon1 | Serum deprivation-response protein | 0.00 | 0.00 | 0.00 | 0.00 |
| Q923D4 | Sf3b5 | Spermine oxidase | 0.00 | 0.00 | 0.00 | 0.00 |
| Q8BND5 | Qsox1 | Splicing factor 3B subunit 5 | 0.00 | 0.00 | 0.00 | 0.00 |
| Q80YX1 | Tnc | TBC1 domain family member 10A | 0.00 | 0.00 | 0.00 | 0.00 |
| P35441 | Thbs1 | Thioredoxin | 0.00 | 0.00 | 0.00 | 0.00 |
| P40142 | Tkt | Transforming growth factor beta-1 | 0.00 | 0.00 | 0.00 | 0.00 |
| P07309 | Ttr | Translocon-associated protein subunit alpha | 0.00 | 0.00 | 0.00 | 0.00 |
| Q06806 | Tie1 | Tropomyosin alpha-3 chain | 0.00 | 0.00 | 0.00 | 0.00 |
| P61089 | Ube2n | Tyrosine-protein kinase receptor Tie-1 | 0.00 | 0.00 | 0.00 | 0.00 |
| Q64339 | Isg15 | Ubiquitin-conjugating enzyme E2 N | 0.00 | 0.00 | 0.00 | 0.00 |
| O88342 | Wdr1 | WD repeat-containing protein 1 | 0.00 | 0.00 | 0.00 | 0.00 |
